# AI-Driven Intrusion Detection for Vehicular Networks: A Comprehensive Survey of Techniques, Datasets, Deployment Architectures, and Future Directions

**DOI:** 10.3390/s26196211

**Published:** 2026-09-30

**Authors:** Syed Rizwan Hassan, Sadia Din, Muhammad Ismail Mohmand

**Affiliations:** 1 Department of Computer Engineering, Gachon University, Seongnam-si 13120, Republic of Korea; syed9919@gachon.ac.kr; 2 School of Computer Engineering, Faculty of Engineering and Natural Sciences, Istanbul Atlas University, Istanbul 34408, Turkey

**Keywords:** intelligent transportation systems, vehicle-to-everything, VANETs, Internet of Vehicles, artificial intelligence, 6G

## Abstract

Intelligent transportation systems (ITSs), Vehicle-to-Everything (V2X) communication and autonomous driving technologies have brought about significant changes in the modern vehicular network. At the same time, the cyber-attack surface has grown, leading to new and existing advanced security threats for Vehicular Ad hoc Networks (VANETs) and the Internet of Vehicles (IoV). Traditional IDSs that employ static rule-based techniques are often insufficient in the context of the vehicular environment, characterized by dynamic, highly mobile systems with stringent low-latency requirements. Next generation IDS development has shifted to artificial intelligence (AI), which offers adaptive, scalable and data-driven detection capabilities. In this survey, the authors present a comprehensive overview of the state-of-the-art of AI-driven vehicle IDS in terms of machine learning (ML), deep learning (DL), federated learning (FL), reinforcement learning (RL), transformer-based techniques, and graph neural network (GNN) designs. We present a multidimensional taxonomy of vehicular attacks, an analysis of existing datasets and their weaknesses, an evaluation of the performance of AI techniques and a study of potential deployment architectures such as edge, fog and cloud. Furthermore, some of the most important open problems are identified, including data scarcity, adversarial robustness, real-time constraints, data utility-privacy conflicts, and standardization gaps. A research roadmap is provided, including 6G connectivity, blockchain-based IDS, explainable AI (XAI), Digital Twin technologies and post-quantum cryptography. This survey provides a common reference for the designers and developers of secure, efficient and interoperable VIDSs.

## 1. Introduction

The evolution of vehicular networks from mechanical to highly connected, cyber-physical environments brings together three domains, namely Vehicular Ad hoc Networks (VANETs), Internet of Vehicles (IoV), and Vehicle-to-Everything (V2X) communication to support sophisticated intelligent transport systems (ITSs) for advanced applications such as flexible collision avoidance, adaptive traffic management, and autonomous driving assistance [1]. Advancements in artificial intelligence (AI) have paved the way for the development of intelligent and adaptive vehicular systems capable of coping with complex communication environments [2]. With the development of vehicular communication technologies such as Dedicated Short-Range Communications (DSRC), Cellular Vehicle-to-Everything (C-V2X), and upcoming 5G-based V2X, vehicle-to-vehicle (V2V), vehicle-to-infrastructure (V2I), and vehicle-to-cloud communication have been greatly improved [3]. All of these technologies will enable efficient communication between vehicles and enable connected vehicles to be widely distributed. The complex nature of integrating diverse communication protocols and architectures makes the vehicular network particularly vulnerable to cyberattacks [4].

Recent developments in vehicular networks have led to the widespread adoption of AI techniques in security [5]. Significant vehicular network problems, such as dynamic network topology, high mobility and large-scale data processing, have been solved using these techniques [6]. An AI-based IDS architecture has been shown to be effective for detecting abnormal behaviours and cyber threats in vehicles equipped with V2X technology [7]. With these advancements, vehicular communication technology has grown more complex, and networks are now vulnerable to attacks, such as spoofing, denial-of-service, and data manipulation [8]. These challenges are further magnified by the decentralized nature of future communication technologies [9]. Moreover, new security issues arise related to network slicing, communication reliability and resource allocation in cellular-based V2X systems [10]. IDSs are among the key technologies used to monitor and mitigate attacks on vehicular networks by analysing traffic patterns and identifying suspicious behaviours [11]. It has been shown that traditional IDSs that use preconfigured signatures and rule sets are insufficient to address the dynamic environment in which a vehicle operates [12]. These systems, however, cannot detect zero-day attacks and can suffer from high false alarm rates due to the diversity of vehicle traffic [13]. To overcome these limitations, AI-based approaches have become increasingly popular in intrusion detection for vehicular networks [14]. In complex environments, an IDS could be further improved by leveraging machine learning and deep learning, enabling automatic feature extraction, adaptive learning, and real-time threat detection [15].

Furthermore, as deep learning architectures become more sophisticated, IDS can now detect multi-stage, complex cyberattacks within the smart car system [16]. In addition, new paradigms such as federated learning, distributed computing and edge computing have facilitated privacy while maintaining security [17]. To address privacy and communication overhead issues, these techniques enable cooperative model training across independent, distributed vehicle nodes. These real-time, low-latency solutions can be complemented by AI-IDS and advanced communication technologies such as 5G and future V2X systems [18]. Based on existing survey work, some gaps remain in AI-based intrusion detection research. A number of surveys focus on detecting intrusions into a network but do not address problems posed by vehicles or specific topics, such as the in-vehicle network or machine learning approaches [19]. Moreover, AI-based IDSs in vehicular networks require a comprehensive re-evaluation of their ability to respond to new attacks. Therefore, current research aims to provide a structured, in-depth analysis of the vehicular network-based AI-IDS system. It explains the existing datasets and methods and provides an overview of future research directions and challenges in secure and smart vehicular communication systems. Table 1 compares the scope of this survey with existing review studies. To ensure an objective comparison, each survey was evaluated using a predefined coding rule. The symbol ✓ indicates comprehensive coverage through a dedicated section or subsection, discussion of at least three relevant studies, and comparative synthesis through a table, taxonomy, or critical analysis. The symbol ∼ indicates partial coverage, where the topic is briefly discussed, supported by only one or two studies, or presented without comparative analysis. The symbol × indicates that the topic is absent or mentioned only incidentally. These criteria were applied consistently to the full text of each survey.

The central novelty of this survey is not the isolated discussion of individual topics, but their integration into a unified evidence-to-deployment perspective. It connects vehicular attack characteristics, dataset suitability, AI-model selection, adversarial robustness, explainability, privacy, and edge/fog/cloud deployment requirements. This cross-sectional synthesis reveals how choices at one stage, particularly dataset selection and model complexity, affect real-time performance, generalization, privacy, and deployment feasibility. The survey also extends existing taxonomies by incorporating agentic-AI, LLM-enabled, physical-layer, sensor-spoofing, and ECU firmware threats.

This survey is motivated by critical questions of pressing interest that are not addressed in the current literature. First, existing research has not provided a consistent, multidimensional taxonomy that accounts for the latest developments in vehicular IDS, such as detection methods, deployment architectures and technologies. Secondly, there is a lack of systematic analysis of vehicle datasets and their appropriateness for realistic evaluation. Thirdly, important research topics such as federated learning, blockchain, explainable AI (XAI), and adversarial robustness are discussed, but their mutual integration is not discussed. Lastly, the implementation issues associated with real-time AI-powered IDS in dynamic, heterogeneous vehicular environments remain underexplored. The major contributions of their comprehensive review are as follows.

- This survey suggests a consolidated multidimensional classification of vehicular IDS that combines detection techniques, deployment architectures, data sources and AI-based techniques.
- It offers a systematic analysis of datasets used for the vehicles themselves and general datasets, and provides an indication of their reliability and utility for realistic IDS development.
- A detailed taxonomy of attacks is provided, mapping to security threats, the CIA requirements, the layers of vehicular architecture, and vulnerabilities specific to AI.
- A comprehensive performance of several major techniques, such as ML, DL, FL, RL, transformer, and GNN models across a range of evaluation metrics is described.
- This study evaluates how the federated learning and blockchain paradigms can be integrated to support IDS systems that meet privacy and security requirements in vehicular networks.
- In conclusion, a forward-looking research roadmap is provided, covering emerging topics such as 6G-enabled V2X, XAI, Digital Twins, post-quantum security, and standardization issues.

### Review Methodology

This study follows a structured narrative-review methodology. Relevant literature was identified through IEEE Xplore, Scopus, Web of Science, ACM Digital Library, ScienceDirect, SpringerLink, and Google Scholar. The search primarily covered studies published between January 2020 and August 2026, while selected earlier studies were retained to explain foundational vehicular-network, communication, and IDS concepts. The search combined the following terms: (“vehicular network” OR “VANET” OR “Internet of Vehicles” OR “IoV” OR “V2X” OR “in-vehicle network”) AND (“intrusion detection” OR “IDS” OR “cybersecurity”) AND (“artificial intelligence” OR “machine learning” OR “deep learning” OR “federated learning” OR “reinforcement learning” OR “transformer” OR “graph neural network” OR “large language model” OR “agentic AI”). The search syntax was adjusted according to the requirements of each database. Backward and forward citation searches were also conducted to identify relevant studies not captured by the initial queries.

Studies were included if they addressed intrusion detection or cybersecurity in vehicular environments and provided relevant information about AI techniques, datasets, attack types, evaluation metrics, or deployment architectures. Studies were excluded if they were duplicated, unavailable in English, lacked sufficient technical information, or had no clear relevance to vehicular security. Generic network and IoT IDS studies were retained only when they provided transferable methodological insights and were clearly distinguished from vehicular-specific evidence. Standards, technical reports, and selected preprints were used mainly to support emerging topics for which limited peer-reviewed evidence was available.

The final evidence base comprised 189 sources, including vehicular IDS studies, related surveys, dataset papers, technical standards, and selected foundational works. Comprehensiveness was assessed using predefined coverage dimensions: vehicular attack categories, AI techniques, datasets, performance metrics, deployment architectures, privacy, adversarial robustness, explainability, blockchain integration, 5G/6G connectivity, and emerging agentic-AI threats. The search was considered sufficiently comprehensive when additional database and citation searches produced no new major methodological, dataset, attack, or deployment category.

## 2. Fundamentals of Vehicular Networks and Security

Vehicular networks are a specialized class of mobile ad hoc networks (MANETs) designed to meet the unique requirements of vehicular communications, comprising vehicles, roadside units and servers. To create effective AI-driven IDS solutions, it is essential to understand their architectural design, communication mechanisms, and security considerations.

### 2.1. Architecture of Vehicular Networks

Vehicular network architecture is built around the Vehicle-to-Everything (V2X) paradigm in which communications can be established among vehicles, infrastructure, pedestrians and cloud servers. V2X is a multimodal communication system that unites several technologies such as V2V, V2I, V2P, V2N and V2C, creating a dynamic, distributed communication network that integrates many communication technologies with high mobility and low latency requirements [13]. Such characteristics pose significant challenges for monitoring and detecting intrusions. Khalil et al. [10] proposed an AI-based IDS framework for V2V communication in VANETs, which uses an on-board unit (OBU) for exchanging safety-critical messages. They use machine learning-based anomaly detection to identify patterns of malicious communication, achieving high detection accuracy in dynamic vehicular environments. This demonstrates the need to embed intelligent IDS systems into the vehicular communication layer.

On the communication side, Tahir et al. [11] have shown that V2V and V2I systems can significantly enhance connectivity and reliability, but may also pose new threats, such as content sorting and copyright infringement, through the open wireless medium. Similarly, Malik et al. [12] stated that introducing V2P communication systems to improve pedestrian safety creates new point-to-point attack surfaces that adversaries can exploit to unfairly modify safety information or send false alarms. The software-defined V2X architecture was introduced at the architectural level by most researchers, including He et al. [14], who used SDN and 5G communication technologies. They enable resources to be allocated on demand and managed from a single location, thus improving network efficiency, but they also increase the network’s vulnerability to attacks such as control-plane manipulation and DDoS. The results in this paper demonstrate that the typical architecture of modern vehicles is multi-layered and must be adapted by IDS solutions that can cover different communication forms. Table 2 summarizes various vehicular network architectures presented in the literature.

Overall, the combination of IoV, edge computing and vehicular software-defined vehicle (SDV) paradigms has resulted in the emergence of a complex cyber-physical system. The developments introduced numerous attack surfaces across the system’s perception, communication, network and application layers, along with many layers of functionality and scalability. Therefore, it is crucial to deploy a layered, intelligent IDS system to enable secure, reliable vehicle communication.

### 2.2. Communication Technologies

**Figure 1:**
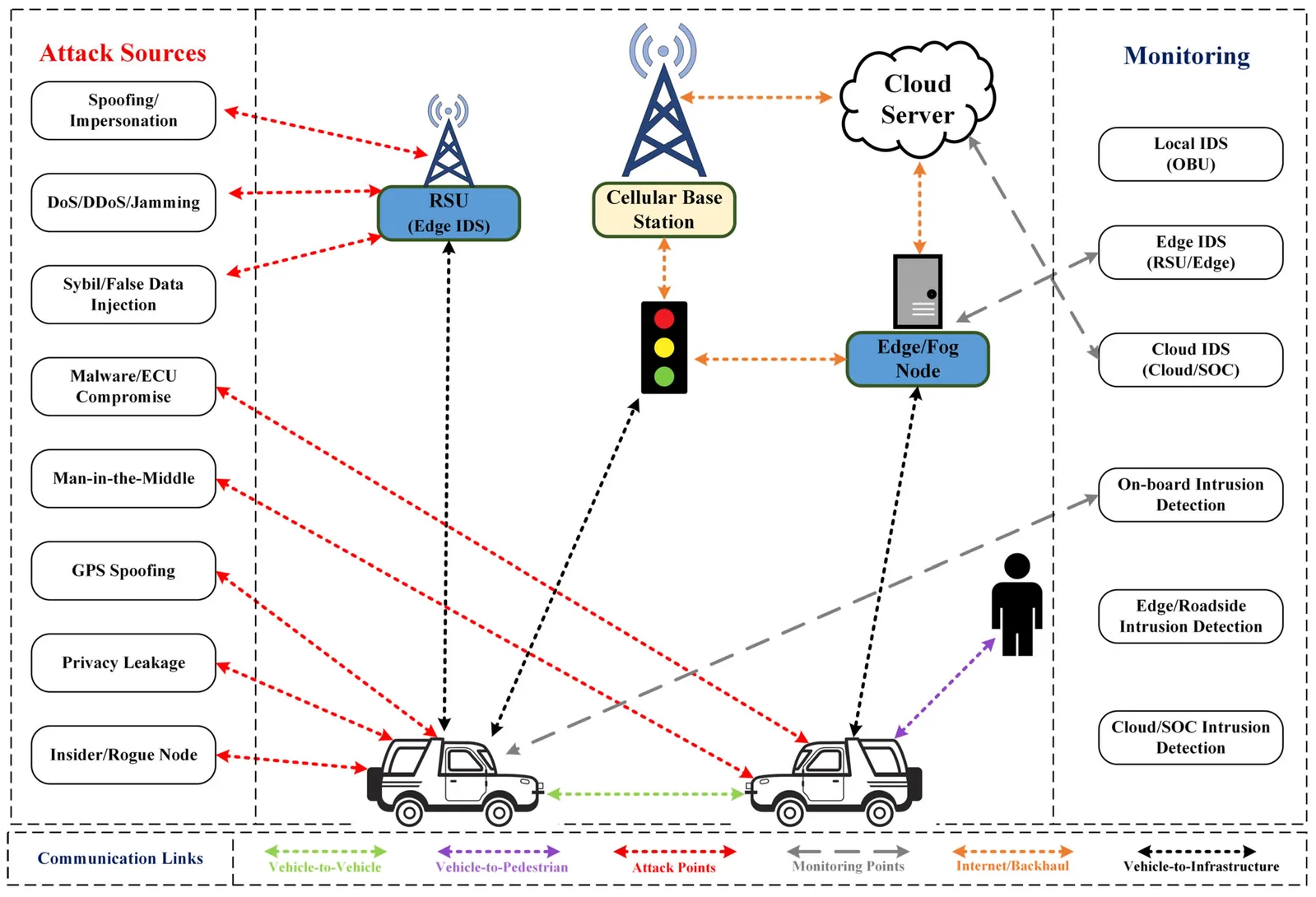
V2X attack surface and AI-IDS monitoring points.

Vehicular communication technologies enable real-time data exchange between various components of vehicular networks. Chen et al. [15] showed that C-V2X, with supported direct (PC5) and network-based communication, also offers additional benefits in terms of channel coverage, reliability and scalability compared to DSRC. Further, Khan et al. [16] show that advanced C-V2X protocols enable ultra-reliable low-latency communication (URLLC), and yet there are other problems, such as spectrum management and interference. Pawar et al. [17] demonstrated that 5G-V2X technology can provide real-time support for vehicular applications with sub-millisecond latency and massive connectivity, but it also presents new security challenges due to edge computing. Similarly, Kandu et al. [18] identified problems in dynamic vehicular environments, including congestion, handover delays and security issues. In LTE/5G-V2X, the research in [19] proposed efficient resource allocation strategies that enhance system performance while allowing for resource manipulation attacks. Authors in [20] explored that future 5G/6G systems with V2X will need to address new cyber threats and require more advanced cyber threat intelligence and adaptive IDS solutions. Table 3 summarized the communication technologies used in recent studies on vehicular networks. Figure 1 describes that modern vehicular networks integrate V2V, V2I, V2P, V2N, and V2C communication, which expands the attack surface across vehicles, RSUs, edge nodes, and cloud infrastructure.

### 2.3. Security Requirements

In addition to the traditional CIA (confidentiality, integrity and availability) Security Triad, vehicular network security also considers other essential elements such as authentication, privacy, non-repudiation and freshness. The requirements for such a system for connected vehicle communications are that the environments in which communications take place are dynamic and highly mobile, and are not sufficiently secure under traditional security assumptions [21]. Palanichamy [22] showed that to ensure systems based on cooperative awareness messaging (CAM) are not susceptible to false data injection and replay attacks, strong integrity and authenticity must be ensured through security systems. Likewise, the security of sensor data and the timeliness of communication in safety-critical vehicular applications are extremely important and are sensitive to integrity violations and availability failures [23]. In connected vehicular networks, safety applications have demonstrated that authentication and freshness are necessary to guarantee that sent messages are authentic and timely [24]. Moreover, Almutairi [25] explored IoT-enabled vehicular networks and found that data exchange and tracking raise serious privacy and confidentiality issues. The study in [26] provides a comprehensive review of security and privacy solutions in VANETs; they noted that “decentralized control of trust, pseudonym solutions and secure communication mechanisms” are necessary to build security and privacy solutions against attacks such as tracking, spoofing, and identity-based attacks. In conclusion, securing vehicular networks is a multidimensional problem that involves upholding the principles of confidentiality, integrity, and availability, as well as advanced principles such as privacy and freshness. These requirements indicate the need for a smart IDS to detect real-time, diverse and changing threats in vehicular networks. Hu and Ma proposed adaptive event-triggered tracking control using switching functions, command-filtered tracking errors, and adaptive uncertainty estimation to ensure finite-time stability under unmodelled dynamics and disturbances [27]. Although control-oriented, this approach provides a useful basis for vehicular IDSs to adapt alert or model-update transmission thresholds according to changing network conditions, thereby reducing unnecessary communication. Table 4 summarizes various vehicular network security requirements and IDS implications.

### 2.4. Threat Model in Vehicular Networks

Vehicular threat models encompass both external and internal threats, each with distinct attack characteristics and capabilities. The interactions between vehicles rely on wireless communication, which can be attacked by external parties and undermine communication reliability. Man et al. [28] demonstrated that external attackers can attack wireless communications in various ways, such as eavesdropping, jamming and message injection, which may severely affect the reliability of wireless communication in IoV systems. Nwakanma et al. [29] noted that today’s vehicle contexts increasingly face individually intelligent and XAI attacks, in which an adversary might influence AI decisions or exploit the context’s “transparency” to hide its actions. Also, Ahmed et al. [30] have shown that the risk of compromised vehicles functioning as an internal attack is extremely high due to the possibility of false data injection, routing interruptions, and the launch of large-scale attacks.

The results of the experiments performed in [31] showed that insider attacks (e.g., compromised ECUs or rogue nodes) are more challenging to detect because the attackers use legitimate credentials and exhibit normal communication behaviour. In addition, Gudekota et al. [32] noted that multi-vehicle attacks, such as distributed denial-of-service (DDoS) and Sybil attacks, complicate detection in connected vehicular networks (CVNs). At training time, Aloraini et al. [33] proposed an adversarial machine learning (AML) attack that can create perturbations to fool IDS systems and demonstrated how it enables exploitation at both training and inference time. During training, adversarial machine learning (AML) attacks are proposed that can cause perturbations to fool IDS systems. The overall vehicular threat landscape is quite dynamic and features multi-layer attacks being carried out using external, internal and AI attacks as described in Table 5. The complexity requires well-designed, intelligent, adaptive and robust IDS systems which can detect both traditional and emerging attacks in real time.

## 3. Intrusion Detection Systems in Vehicular Networks

Vehicular networks possess a suite of security defences that are vital to thwart the wide-ranging, dynamic threat landscape outlined in Section 2. This section provides a systematic overview of the goals, classification and deployment architecture of IDSs for vehicular networks.

### 3.1. IDS Overview and Objectives

Intrusion detection systems are important for vehicular network security because they monitor communication patterns and detect malicious activity. Vehicular IDS differs from traditional IDS by operating in environments with strict constraints, such as real-time detection, limited compute power, and highly dynamic network conditions [34]. A survey demonstrated that a deep learning approach can significantly improve detection performance by automatically extracting complex features from vehicular network traffic. According to the study they conducted, IDS based on AI can handle the high-dimensional datasets produced by CAN bus and V2X communication systems [35].

In addition, Lampe and Meng [36] stated that the problems of high false positives and dataset imbalance are extremely critical and significantly affect the reliability of detection in real environments, posing a challenge for vehicular IDS. Also, the work by Zhang et al. [37] introduced an optimization-based IDS framework that achieves high detection accuracy while maintaining low delay, thereby meeting the requirements of safety-critical vehicular applications. Mehedi et al. [38] presented a deep transfer learning-based IDS for vehicular networks, demonstrating how to leverage pre-trained models to enhance detection accuracy while reducing training overhead. Furthermore, Anbalagan et al. [39] proposed an intelligent IDS system for AVs, which builds an adaptive learning and real-time response mechanism to achieve secure and efficient vehicular communication. The global goal of an IDS system in a vehicle is to hold a balance between accuracy, low latency and low resource usage. The needs outlined above underscore the need for lightweight, intelligent, and adaptive IDS schemes that operate in real time within resource-constrained vehicle networks. Table 6 summarizes the key objectives and performance requirements of IDS in vehicular networks.

### 3.2. Types of Intrusion Detection Systems

#### 3.2.1. Signature-Based Detection

Signature-based intrusion detection matches network activities against pre-determined attack pattern(s). This type of prevention has been shown by Jin et al. [40] to be quite accurate at preventing known CAN bus attacks but ineffective against unknown attacks. Alnasser et al. [41] and Nawaal et al. [42] noted that a signature-based IDS is less computationally intensive to implement in a vehicular network and can be used in resource-limited environments; however, it is vulnerable to zero-day attacks and requires continuous, frequent updates. The feasibility of combining a signature-based technique with adaptive methods to enhance detection performance has been demonstrated by Kwon et al. [43] and Nafea et al. [44], albeit at increased system complexity. Agoramoorthy et al. [45] found that although signature-based IDSs can be used against known attacks, they must be complemented with intelligent approaches to handle unknown and evolving attacks. Overall, these studies demonstrate high accuracy and low overhead, but with the limitation of not detecting novel attacks in a dynamic vehicular environment, as summarized in Table 7.

#### 3.2.2. Anomaly-Based Detection

Anomaly-based IDS uses a technique that trains a normal behaviours model to detect intrusions and identify anomalies. Agrawal et al. [46] demonstrated the efficacy of anomaly detection in defending against in-vehicle networks, detecting both unknown and insider attacks without the need for pre-known signatures. Although machine learning and deep learning models have been shown to be effective for accurate detection in dynamic vehicular environments [47,48], it is not straightforward to determine what constitutes ‘normal’ behaviours in a complex traffic environment. This variation, however, can lead to higher false alarm rates than those observed with signature-based systems [49]. In recent years, to enhance detection robustness, several improvements have been proposed, such as IDS based on graph neural networks [50], which model complex network relationships in vehicular data. The authors in [51] again noted that using more advanced anomaly-detection models, such as autoencoders or generative models, could help minimize false positives by learning more accurate representations of ’normal’ behaviour. Table 8 summarizes these studies and shows that in vehicular environments, anomaly-based IDS has proven effective for detecting unknown attack scenarios but has faced significant challenges due to high false-positive rates and dynamic traffic patterns.

#### 3.2.3. Specification-Based Detection

IDSs based on a specification look for abnormalities in the systems or their transaction behaviours relative to one or more specifications. It has been shown to be successful in cyber-physical systems where normal behaviours can be formally defined, leading to a low false positive rate (FPR) [52]. Structured systems protocol violations can be accurately detected by rule-based IDSs, which necessitate detailed rule design and maintenance [53]. Luo et al. [54] applied this to in-vehicle networks in modern times and demonstrated how specification rules and intelligent models can be combined to enhance detection capability. The author in [55] demonstrated that specification-based IDS is appropriate for well-defined vehicular protocols but is difficult to extend when the vehicle’s system architecture is not standardized. Park et al. [56] and Puder et al. [57] also demonstrated that adding graph-based and pattern-based specifications to the specifications increases the accuracy of the detected specifications, but the increased system complexity and the maintenance workload remain challenges. Table 9 shows that specification-based IDS would be accurate and false-alarm-free when the specification is clearly defined, but its scalability and adaptability would be limited in dynamic, software-defined vehicular environments.

#### 3.2.4. Hybrid IDS Approaches

Hybrid IDS is a fusion of several detection methods to achieve greater accuracy and coverage. Zhang and Ma [58] demonstrated that combining signature-based and anomaly-based detection improves detection accuracy, detecting both known and unknown attacks. To achieve higher detection efficiency, Yang et al. [59] proposed a multi-tier hybrid IDS with a layered architecture. Similarly, Lo et al. [60] proposed a CNN–LSTM hybrid network that incorporates both spatial and temporal features of vehicles to achieve very high accuracy. Hassan et al. [61] and Bangui et al. [62] demonstrated that using feature selection with deep learning and ensemble methods improves detection robustness and reduces dimensionality. Another study by Mehmood et al. [63] found that hybrid ML approaches are more effective than single ML approaches but incur additional computational overhead. The model proposed by Ullah et al. [64] is a hybrid deep learning IDS (HDL-IDS) that achieves high detection accuracy in IoV environments but has high computational requirements. In conclusion, hybrid IDSs offer improved detection capabilities and adaptability; however, they have drawbacks in terms of complexity, computing resources, and deployment in resource-limited environments within the vehicular domain. Table 10 provides summary of hybrid IDS techniques deploymed for vehicular networs.

### 3.3. IDS Deployment Architectures

**Figure 2:**
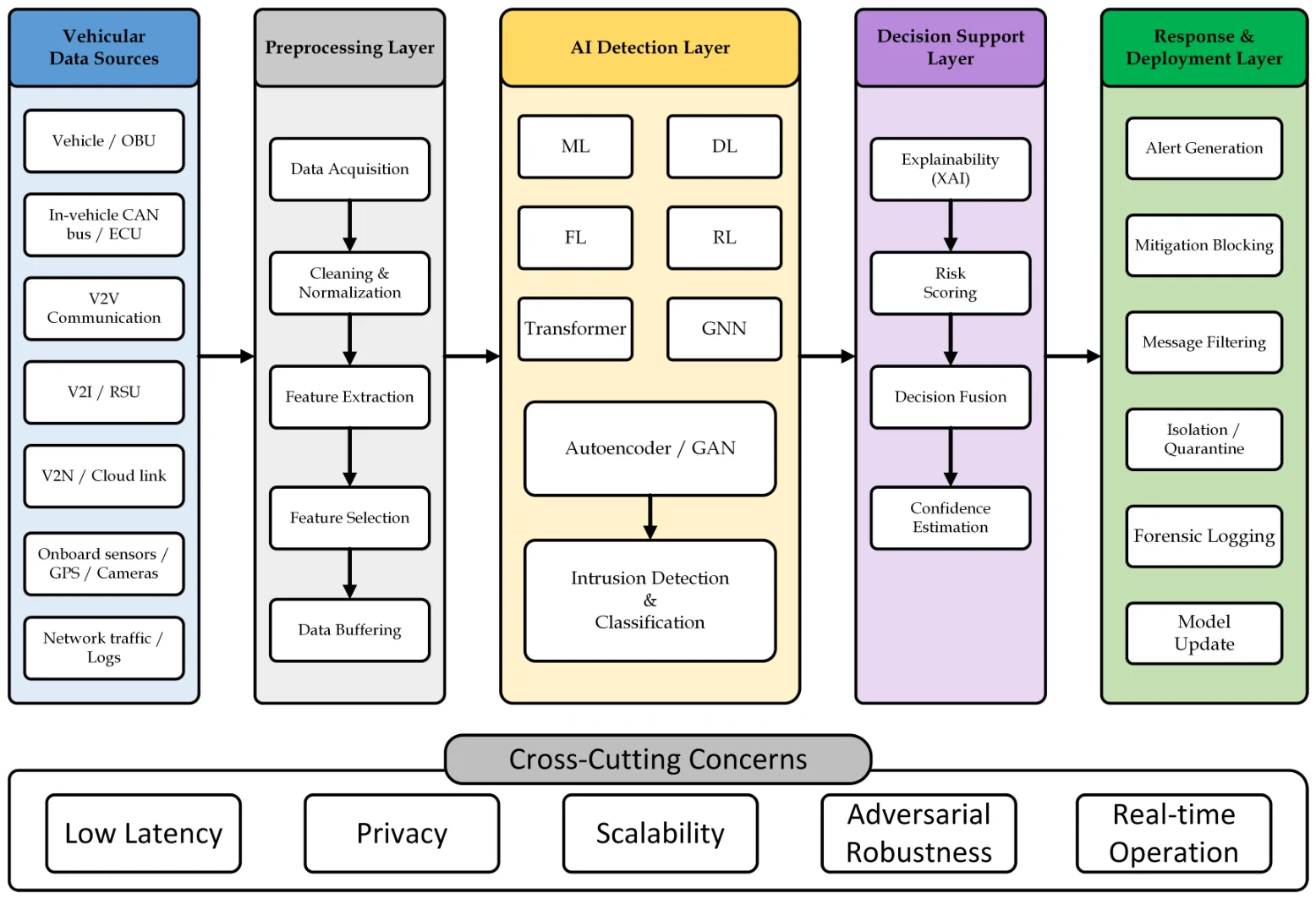
Generalized AI-driven vehicular IDS architectures.

The deployed architecture of the vehicular IDS plays a significant role in factors such as detection latency, scalability and privacy. A centralized IDS was proposed by Ahmad et al. [65], that implements a centralized IDS with a cloud aggregator to detect large-scale attacks, but this approach incurs high latency and communication costs. To enable real-time threat detection, Sorkhpour et al. [66] proposed an autonomous distributed IDS (Auto-CIDS) that enables local detection at vehicle nodes. In a similar fashion, the authors of Song et al. [67] showed that embedding deep learning models in IoTVs can detect with low latency, but are limited by computational resources. Quadar et al. [68] suggested that an IDS based on the edge and an IDS based on automotive Ethernet offer advantages in scalability and component communication delay, but that they pose new challenges related to distributed coordination. Christy et al. [69] proposed a multi-stage hierarchical IDS based on a cloud-overlay vehicular layered architecture to achieve a high detection rate while handling greater scalability. El-Fatyany et al. [70] noted that hybrid and federated architectures are desirable solutions for balancing competitors’ privacy and performance, with the detection task divided among vehicles, RSUs and the cloud platform. Desai and Chatterjee [71] also showed that having real-time IDS at the edge of their networks improves predictive threat detection, but the resources must be managed efficiently. In summary, centralized architectures can have strong analytical capabilities, while distributed or hybrid architectures can provide high privacy and low latency. Today, vehicular IDS is increasingly using hierarchical and edge-based systems to meet the requirements of performance, scalability and real-time detection. Table 11 summarizes these deployment architectures. The generalized AI-driven vehicular IDS architecture in Figure 2 summarizes how raw vehicular data are processed through feature extraction, AI-based detection, explainable decision support, and automated response across OBU, RSU/fog, and cloud tiers.

In high-mobility environments, frequent handovers between RSUs or edge servers can cause interference, packet loss, latency, and fragmented traffic observations, reducing IDS reliability. Edge-based IDSs should therefore employ mobility-aware handover prediction, multi-connectivity, rapid IDS-state migration, and cross-edge coordination to maintain continuous detection.

Vehicular IDS placement should depend on the application latency budget. Safety-critical in-vehicle functions require approximately 1–10 ms detection and should execute lightweight monitoring, inference, and emergency response locally on the ECU/OBU. Cooperative V2X safety applications generally require 10–50 ms; therefore, local detection should be combined with RSU/MEC-based multi-vehicle correlation. Traffic management and non-critical services can tolerate approximately 50–100 ms and may be processed at the edge. Cloud platforms are more appropriate for delay-tolerant functions exceeding 100 ms, including model training, long-term analysis, threat-intelligence storage, and global model updates. Thus, hierarchical deployment provides the most practical balance between latency, privacy, and computational capacity.

### 3.4. Implementation and Deployment Workflow for AI-Based Vehicular IDS

Implementation begins by defining the attack scope, monitored vehicle components, deployment location, and operational requirements. Data are then collected from CAN buses, automotive Ethernet, OBUs, sensors, RSUs, and V2X interfaces. The collected data should be cleaned, synchronized, labelled, balanced, and divided into independent training, validation, and testing sets. The AI model is subsequently selected according to the attack type, data modality, explainability needs, and available computational resources.

Before deployment, the model should be evaluated using detection accuracy, precision, recall, F1-score, false alarm rate, inference latency, memory consumption, energy usage, and adversarial robustness. Hardware-aware optimization methods, including pruning, quantization, and knowledge distillation, can be applied when deploying complex models on resource-constrained OBUs. The optimized model should also be tested using realistic mobility, communication-delay, packet-loss, and previously unseen attack conditions.

Model placement depends on operational requirements. OBU-based deployment provides immediate local detection, RSU or fog deployment supports collaborative regional analysis, and cloud deployment enables computationally intensive training and global model management. A hybrid architecture can distribute inference across these layers according to latency and privacy requirements. After deployment, data drift, prediction confidence, false alarms, resource consumption, and emerging attacks should be continuously monitored. Models should be updated through authenticated and integrity-protected over-the-air mechanisms with version control, rollback support, and human approval for safety-critical actions.

The essential AI requirements include representative datasets, sufficient processing and memory resources, low inference latency, model robustness, privacy protection, explainability, secure model storage, authenticated updates, and continuous lifecycle monitoring.

## 4. AI Techniques for Vehicular Intrusion Detection

From basic statistical anomaly detection to complex deep learning architectures, federated learning frameworks, and transformer-based semantic models, the adoption of AI for vehicular IDS has advanced by leaps and bounds, moving from rudimentary to advanced methods. The journey of AI in vehicular IDS has made a huge leap from the early days of statistical approaches to the complex architectures of deep learning, federated learning, and transformer-based semantic models. This section offers detailed overviews of the major AI technique categories, highlighting the foundations of algorithms, how they have been adapted for vehicles, sample applications, and performance considerations. To distinguish vehicular-specific evidence from general IDS literature, the studies were classified according to their evaluation environments. Of the 54 studies in Table 12, Table 13, Table 14, Table 15, Table 16, Table 17, Table 18, Table 19 and Table 20, 11 (20.4%) use vehicular datasets or scenarios, including CAN, VANET, IoV, ITS, connected-vehicle data, and vehicular simulations. The remaining generic IoT, IIoT, WSN, ICS, and network studies are included only to explain transferable AI techniques and are not treated as direct validation of vehicular IDS performance.

### 4.1. Machine Learning-Based IDS

#### 4.1.1. Supervised Learning Approaches

In vehicular IDS, supervised machine learning techniques are popular because they can be well trained on labelled data, yielding high accuracy and interpretability. To highlight the benefits of supervised learning models for IDS, Baniasadi et al. [72] showcased that supervised learning approaches can be effective in detecting intrusions in an IoT network using labelled IoT traffic features. Kumar et al. [73] and Chenniappanadar et al. [74] demonstrated the application of classical algorithms, such as Random Forest and SVM, achieving high detection accuracy with minimal computational requirements, making them apt for a vehicular environment. Navaneetha et al. [75] also showed that supervised learning-based approaches are effective when combined with others to better detect under different attack scenarios. However, Alshahrani et al. [76] also noted that supervised IDSs are susceptible to adversarial attacks, which can be applied to the IDS’s input to evade detection. Likewise, Rani and Kaushal [77] highlighted the need for labelled datasets and noted difficulties with class imbalance and unexpected attack patterns. Overall, supervised learning methods are very accurate for attacks defined in their training sets, but suffer from data dependence and problems due to a lack of labels or coverage.

#### 4.1.2. Unsupervised Learning Approaches

Unsupervised learning methods are suitable for vehicular environments, where there is little labelled data, because they learn anomalies from their observations. In the study by Sadia et al. [78], it was established that unsupervised ML techniques can effectively detect anomalous network traffic patterns, especially for unknown attacks. Deviations in system behaviour have been shown to be possible using clustering and statistical methods by Choi and Kim [79], whose results rely on a proper definition of normal patterns. Kikissagbe and Adda [80] also noted that the unsupervised model is effective for detecting zero-day attacks, though it is sometimes considered less accurate due to high false-positive rates. Both Rashid et al. [81] and Vikram [82] demonstrated that combining multiple unsupervised techniques achieved high detection accuracy, and Olanrewaju-George and Pranggono [83] showed that integrating unsupervised learning with federated frameworks improved scalability and adaptability in distributed environments. Table 13 describes that the unsupervised IDS can detect unknown attacks but must be carefully tuned to minimize false alarms in networks with high vehicle dynamism.

### 4.2. Deep Learning-Based IDS

#### 4.2.1. Convolutional Neural Network Approaches

The use of Convolutional Neural Networks (CNNs) is an important field in the vehicular IDS, as they are widely used for automatically extracting features from network traffic. To achieve high detection accuracy, Qazi et al. [84] designed a 1D-CNN-based IDS that learns patterns directly from raw traffic data. Al-Turaiki and Altwaijry [85] showed that CNN-based models outperform standard models by computing spatial correlations of network features and can effectively handle complex attack patterns. They also demonstrated that CNN-based models outperform standard models, as they capture spatial correlations in network features and can effectively handle complex attack patterns. Lansky et al. [86] also noted that deep learning models (especially CNNs) are superior to classical ML methods for large-scale intrusion detection. There is also a hybrid option that has proven effective. Qazi et al. [87] proposed a hybrid deep learning IDS based on a CNN, combining it with other models, achieving higher detection accuracy and robustness. Likewise, Khan [88] proposed a CNN–RNN hybrid system to improve network performance in detecting sequential and multi-stage attacks. To account for time sequences, Kasongo [89] demonstrated that recurrent models were suitable for complementing CNN architectures, but at a higher computational cost. Overall, CNN-based IDS offers high accuracy and impressive feature extraction performance, yet it suffers from the drawback of requiring enormous computational resources and real-time implementation in resource-constrained vehicular networks as summarized in Table 14.

#### 4.2.2. LSTM and Recurrent Architectures

Vehicular IDS can greatly benefit from recurrent neural networks, particularly LSTM models, which are particularly useful for modelling temporal dependencies in network traffic. Vinayakumar et al. [90] demonstrated the superior performance of LSTM models over conventional RNNs for detecting intrusion patterns in sequence data. The optimized LSTM model was proposed by Dash et al. [91] and achieved high detection accuracy by optimizing the sequence learning process. Odiaga et al. [92] also showed that the LSTM model outperforms a traditional Deep Neural Network (DNN) in identifying cyberattacks over time. Imrana et al. [93] developed a bidirectional LSTM (BiLSTM) approach that achieved better detection accuracy by incorporating the traffic context from both the past and the future. Kanna and Santhi [94] proposed a hybrid algorithm combining LSTM with feature extraction to achieve higher detection accuracy. These researchers [95] presented a recursive ensemble IDS that demonstrated greater robustness and performance, albeit at the expense of higher computational complexity. Table 15 summarizes all these studies. In contrast, the overall performance of LSTM-based IDS is quite satisfactory for sequential and multi-stage attacks, but has some drawbacks in terms of computation overhead and real-time deployment in vehicular systems.

#### 4.2.3. Hybrid CNN-LSTM and Transformer Architectures

The hybrid CNN–LSTM model is used for intrusion detection, combining spatial and temporal feature learning. Liu et al. [96] proposed a model based on the CNN–LSTM–transformer framework to achieve an excellent detection rate by utilizing both local and sequential flow characteristics. Wang et al. [97] and Polat et al. [98] demonstrated that combining spatial and temporal inputs with a CNN-LSTM model can significantly improve anomaly detection accuracy. Yao et al. [99] also demonstrated that CNN–transformer models outperform traditional model architectures in modelling long-range dependencies. Du et al. [100] and Saleh et al. [101] noted that transformer-based IDS is more accurate at identifying global relationships among network traffic, thereby enhancing its detection accuracy and adaptability. Fares et al. [102] furthered this idea by applying hybrid transformer–LSTM models that, with transfer learning, achieve high performance. Overall, the hybrid CNN–LSTM and transformer-based IDS yields state-of-the-art accuracy with promising feature representation, yet with problems of computational complexity and real-time deployment. Table 16 provides a summary of all these studies.

#### 4.2.4. Graph Neural Networks and Geometric Learning

In vehicular IDS, modelling vehicles and RSUs as nodes and their dynamic interactions as edges yields graphs as an appropriate modelling paradigm. The vehicular IDS modelling situation data graph is an appropriate model, as vehicles and RSUs are modelled as nodes, and interactions are modelled as dynamic edges. In complex systems, Zarnegaro et al. [103] demonstrated that GNN-based IDSs achieve significantly better detection of network traffic when structural relationships are considered. Duan et al. [104] designed a dynamic graph-based IDS based on semi-supervised learning that can model changing network topology and achieve high detection accuracy. Hasan and Nijhum [105] also demonstrated that GNNs can detect distributed attacks in real time by analysing node connectivity patterns. Narasimha Prasad et al. [106] highlight the improvements in anomaly detection in networked systems enabled by geometric deep learning, whereas Villalba-Díez et al. [107] underscore its use in cyber-physical systems, such as vehicular networks. Sirico [108] showed that a graph-based modelling approach enhances understanding of complex interactions at the system level, but introduces computational complexity. GNN-based IDS systems offer robust support for coordinated and topology-based attacks, but they face scalability, computational cost, and real-time deployment challenges. Table 17 summarizes these studies.

**Table 17:** Graph neural network approaches in IDS.

Ref.	Methodology	Model	Key Result	Limitation
[103]	GNN-based IDS	Graph Neural Network	Improved detection using graph structure	Computational overhead
[108]	Graph-based system modelling	Geometric DL	Enhanced system-level analysis	High complexity
[104]	Dynamic graph IDS	Graph-based semi- supervised model	Accurate detection in evolving networks	Training complexity
[105]	Real-time IDS	GNN-based model	Effective distributed attack detection	Resource intensive
[106]	Geometric DL IDS	Graph-based anomaly detection	Improved anomaly detection	Scalability issues
[107]	Cyber-physical IDS	Geometric deep learning	Strong modelling of complex systems	High computational cost

**Table 18:** Federated learning-based IDS approaches.

Ref.	Framework	Dataset	Accuracy	Privacy Mechanism	Limitation
[109]	FL-based IDS	IoV data	High accuracy	Local data training	Communication overhead
[110]	FL + Blockchain	Vehicular edge data	Improved detection	Blockchain + FL	Increased complexity
[111]	FL-based IDS	IoV dataset	High performance	Data privacy preservation	Convergence issues
[112]	Edge-based FL-IDS	Transportation IoT	Scalable detection	Local training	Resource constraints
[113]	FL + XAI	Connected vehicle data	Transparent detection	Explainable AI + FL	Computational overhead
[114]	FL-based VANET IDS	VANET dataset	Effective intrusion detection	Distributed learning	Partial participation issues

**Table 19:** Reinforcement learning-based IDS approaches.

Ref.	Methodology	Model	Key Result	Dataset	Limitation
[115]	Multi-agent RL IDS	RL-based multi-agent system	Improved cooperative detection	Network dataset	Training complexity
[116]	Multi-agent IDS survey	RL-based models	Enhanced decentralized detection	Multiple datasets	Implementation complexity
[117]	RL for autonomous systems	Multi-agent RL	Adaptive decision-making	Vehicular simulation	High computational cost
[118]	Decentralized RL IDS	Multi-agent RL	Efficient distributed detection	Network data	Convergence issues
[119]	Cooperative RL system	Multi-agent RL	Improved robustness	Smart city data	Resource intensive
[120]	Few-shot RL IDS	Multi-agent RL	Rapid adaptation to new attacks	VANET dataset	Limited scalability

**Table 20:** Generative and autoencoder-based IDS approaches.

Ref.	Methodology	Model	Key Result	Dataset	Limitation
[121]	GAN-based IDS	Generative Adversarial Network	Improved detection via data augmentation	Network dataset	Training instability
[122]	VAE-based IDS	Variational Autoencoder	Effective anomaly detection	VANET dataset	Reconstruction limitations
[123]	Autoencoder IDS	LSTM Autoencoder	Detects temporal anomalies	ITS data	Computational overhead
[124]	IDS survey	Generative DL models	Improved detection of rare attacks	Multiple datasets	Implementation complexity
[125]	Deep learning IDS	Generative models	Enhanced robustness	Vehicular data	High training cost

### 4.3. Federated Learning for Vehicular IDS

Federated learning (FL) enables collaborative learning across vehicles without sharing raw data, preserving the privacy of IDS training in vehicular networks. The IDS based on FL can successfully learn the attack patterns while maintaining data locality, as shown by Yang et al. [109]. Abou El Houda et al. [110] combined the capabilities of blockchain and FL to improve vehicular edge environments, where collaborative intrusion and detection must be trusted and secure. Huang et al. [111] have presented an IDS strategy based on FL to address privacy concerns in IoV systems and demonstrated high detection accuracy. To efficiently and cost-effectively detect intrusions in the transportation IoT network, Bhavsar et al. [112] proposed an edge-based FL-IDS. To enhance the transparency and trust in IDS decisions, Taheri and colleagues [113] introduced explainable AI into the FL framework. In addition, Arya et al. [114] demonstrated that an IDS based on FL can detect intrusion attacks in dynamic VANET environments. In sum, while both FL-based IDS provide good privacy preservation and scalability, they also incur communication overhead, partial participation and application model convergence issues in highly dynamic vehicular environments. Table 18 summarizes all these studies.

### 4.4. Reinforcement Learning and Multi-Agent Approaches

Adaptive intrusion detection can be achieved through reinforcement learning (RL), which tailor’s optimal policies to the vehicular environment. Tellache et al. [115] proposed intrusion detection using multi-agent RL, with nodes distributed across the network collaborating to detect intrusions. The results presented by Saeed et al. [116] and Dinneweth et al. [117] highlighted that multi-agent systems can help achieve these improvements in target acquisition in a dynamic environment by distributing decision-making among agents rather than centralizing it. Louati et al. [118] proposed an IDS based on decentralized RL and achieved high detection effectiveness in a distributed network. Their further work also demonstrated that cooperative RL can enhance system stability in a smart vehicular environment [119]. Shafiq et al. [120] proffered a multi-agent few-shot IDS that is able to learn quickly and adapt to new attack patterns from small quantities of data. To sum up, some disadvantages of RL-based IDSs include: training complexity, convergence time and real-time implementation in vehicular systems as described in Table 19.

### 4.5. Generative and Autoencoder-Based Approaches

Many generative models and Autoencoders are employed in vehicular IDS for anomaly detection and data augmentation. To improve detection performance, Park et al. [121] showed that using GAN-based IDS to generate realistic attack samples can help. Vehicular behaviour was found to be effectively modelled by variational autoencoders (VAEs) and can be accurately detected from anomalies by Nissar et al. [122]. Likewise, Ashraf et al. [123] presented an LSTM autoencoder model that achieved impressive results in detecting anomalies in intelligent transportation systems. Generative approaches have demonstrated that they increase data diversity and, thus, help detect rare or zero-day attacks, as noted by Khan et al. [124]. Furthermore, Aggarwal [125] has demonstrated that deep generative models can enhance the robustness of an IDS but also increase system complexity. In summary, the generative IDS and autoencoder-based IDS offer valuable capabilities for anomaly detection and data augmentation, respectively, but come with implementation challenges such as training complexity and real-time deployment as described in Table 20.

## 5. Datasets for AI-Based Vehicular IDS

The performance of AI-based vehicular IDS heavily relies on the quality, representativeness, and vehicular-specific nature of the training and evaluation data. In this part, a systematic study of publicly available datasets used in vehicular IDS research has been conducted, focusing on the nature of the datasets, their relevance to the vehicular domain, and the critical weaknesses in their use.

### 5.1. Traditional IT and Network Security Datasets

Traditional datasets are widely used in IDS research, but are limited in their applicability to vehicular environments. However, authors in [126] noted that the attack patterns in legacy datasets, such as KDD Cup 99 and NSL-KDD, are outdated and do not reflect the diversity of real-world traffic, hindering effective modern IDS evaluation. However, Abreu et al. [127] stated that the absence of vehicular-specific protocols, e.g., CAN and V2X, in these datasets renders them irrelevant for automotive security. Guerra et al. [128] moreover showed that using auto-related datasets for training IDS models results in significant performance limitations when these models are deployed in vehicular scenarios. Shafique et al. [129] demonstrated that real-world datasets must be more realistic and domain-specific for modern AI-driven attacks, while Abuali et al. [130] noted that attack datasets such as CICIDS2017/2018 feature more diverse attacks but lack vehicular communication characteristics. Based on this, traditional network security datasets are useful for benchmarking, but the lack of mobility patterns, V2X protocols and in-vehicle communication datasets makes them insufficient for evaluating the performance of the in-vehicle IDS. Table 21 summarizes these dataset studies.

### 5.2. Vehicular-Specific Datasets

While traditional datasets do not include V2X communication patterns, vehicular-specific datasets are necessary and helpful for realistic IDS evaluation. Users of IDS can achieve better performance by training the system with domain-specific data and deploying it within a system that includes realistic attacks, as proposed by Khan et al. [131], while accounting for the vehicles’ context. Vijayakumar et al. [132] created a VANET-based dataset for deep learning-based intrusion detection and localization, achieving improved detection accuracy in the vehicular environment. Similarly, Jayakrishna and Prasanth [133] created a dataset focused on DDoS attacks against VANETs, enabling them to validate the deep learning-based IDS model they developed. Unlike many other benchmarks, such as VeReMi, that offer labelled scenarios of vehicular attacks, most benchmarks are simulated and have limited applicability [134]. Realistic communication patterns and high-end attack scenarios are incorporated into the IDS generalization using new datasets such as PQ-V2X [135]. Taken as a whole, the amount of data available for a particular type of vehicle is a tremendous help to the IDS evaluation but is constrained by simulation limitations, the absence of a standard dataset, and limited coverage of many attack types. Table 22 reveals a clear separation between in-vehicle and external vehicular datasets. Seven of the eleven datasets are CAN-focused, whereas four capture V2X or vehicular communication behaviour; none jointly captures synchronized CAN, V2X, sensor and mobility data. Six datasets contain real CAN traffic, four are simulation-based, and PQ-V2X combines real cryptographic traces with synthetic or hybrid records. Most real datasets use only one vehicle, while can-train-and-test is the principal multi-vehicle benchmark. Significant class imbalance is also evident, particularly in CICIoV2024 and the context-aware VCN dataset. Consequently, high within-dataset accuracy may not indicate robustness across vehicles, communication interfaces or realistic attack prevalence.

### 5.3. Dataset Limitations and Research Gaps

**Figure 3:**
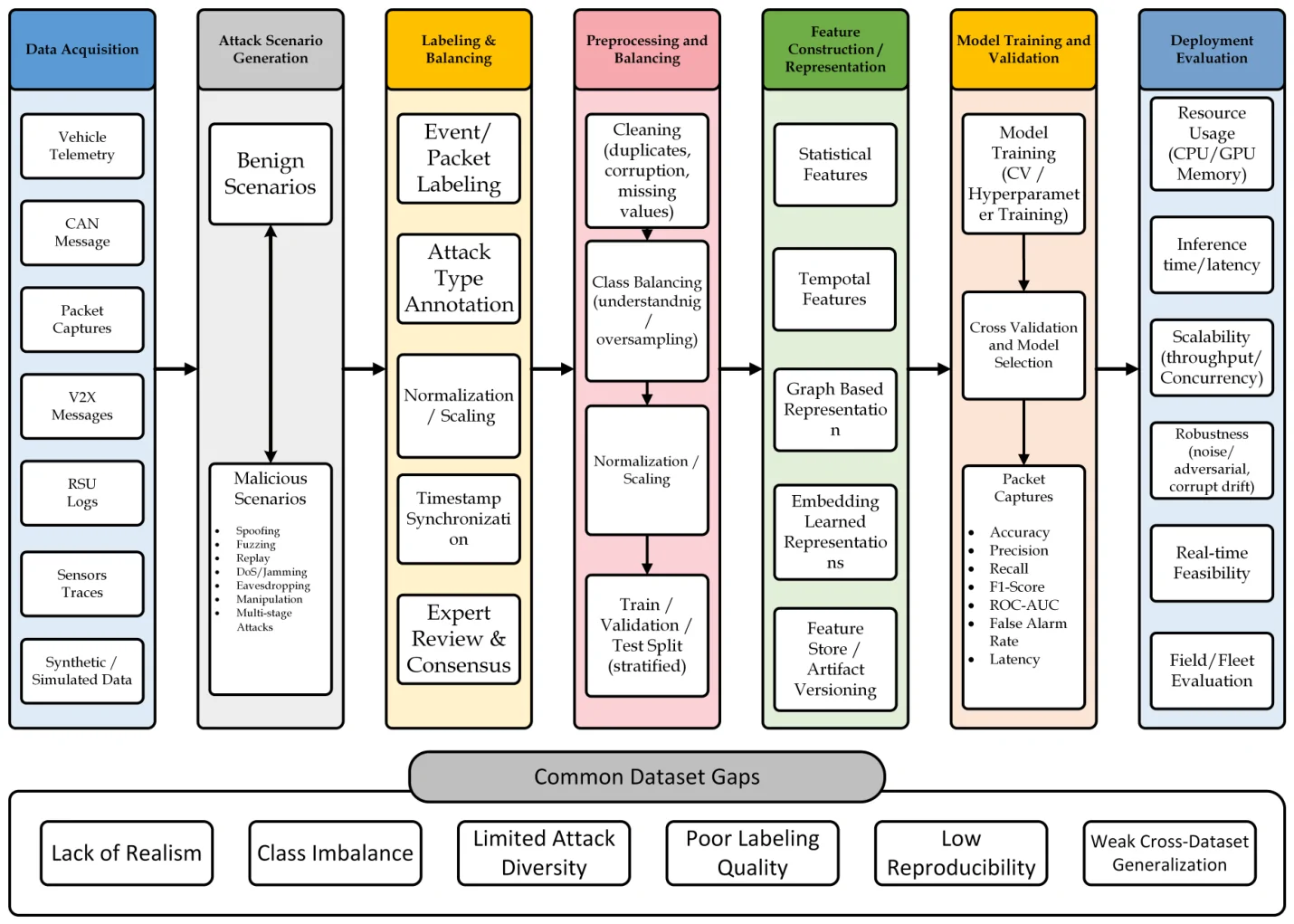
Dataset-to-deployment pipeline for AI-based vehicular IDS.

These traditional datasets are widely used in IDS studies but are limited in their applicability to vehicles. However, Moustafa [126] noted that the attack patterns in legacy datasets, such as KDD Cup 99 and NSL-KDD, are anachronistic and lack the diversity of real-world traffic, hindering the effective evaluation of modern IDSs. Abreu et al. [127], however, stated that the lack of a vehicular-specific protocol in these datasets, such as CAN or V2X, renders them unusable for automotive security analysis. In addition, a study demonstrated that the applicability and performance of using auto-related datasets for training IDS models are very restrictive when the models are used in a vehicular environment [128]. As explained by Shafique et al. [129], real-world datasets should be more realistic, include more relevant attacks and exclude vehicular communication characteristics. Abuali et al. [130] noted that attack datasets such as CICIDS2017/2018 contain more pedestrianized attacks but lack characteristics of vehicular communication. Based on this, traditional network security datasets are useful for benchmarking, but the lack of mobility patterns, V2X protocols and in-vehicle communication datasets makes them insufficient for evaluating the performance of the in-vehicle IDS. Table 23 summarizes these studies, and as summarized in Figure 3, reliable vehicular IDS evaluation requires a complete dataset-to-deployment pipeline, including realistic data acquisition, attack modelling, labelling, balancing, feature construction, validation, and system-level deployment metrics.

The sim-to-real degradation can be quantified as $\Delta F1=F1_{\mathrm{simulation}}-F1_{\mathrm{real}}$ and $\Delta FAR=FAR_{\mathrm{real}}-FAR_{\mathrm{simulation}}$ for the same model. However, most reviewed studies evaluate only one dataset and do not report paired simulation-to-real testing, preventing a reliable numerical meta-comparison. Table 22 distinguishes simulated, laboratory, and real-road datasets.

## 6. Attack Taxonomy in Vehicular Networks

**Figure 4:**
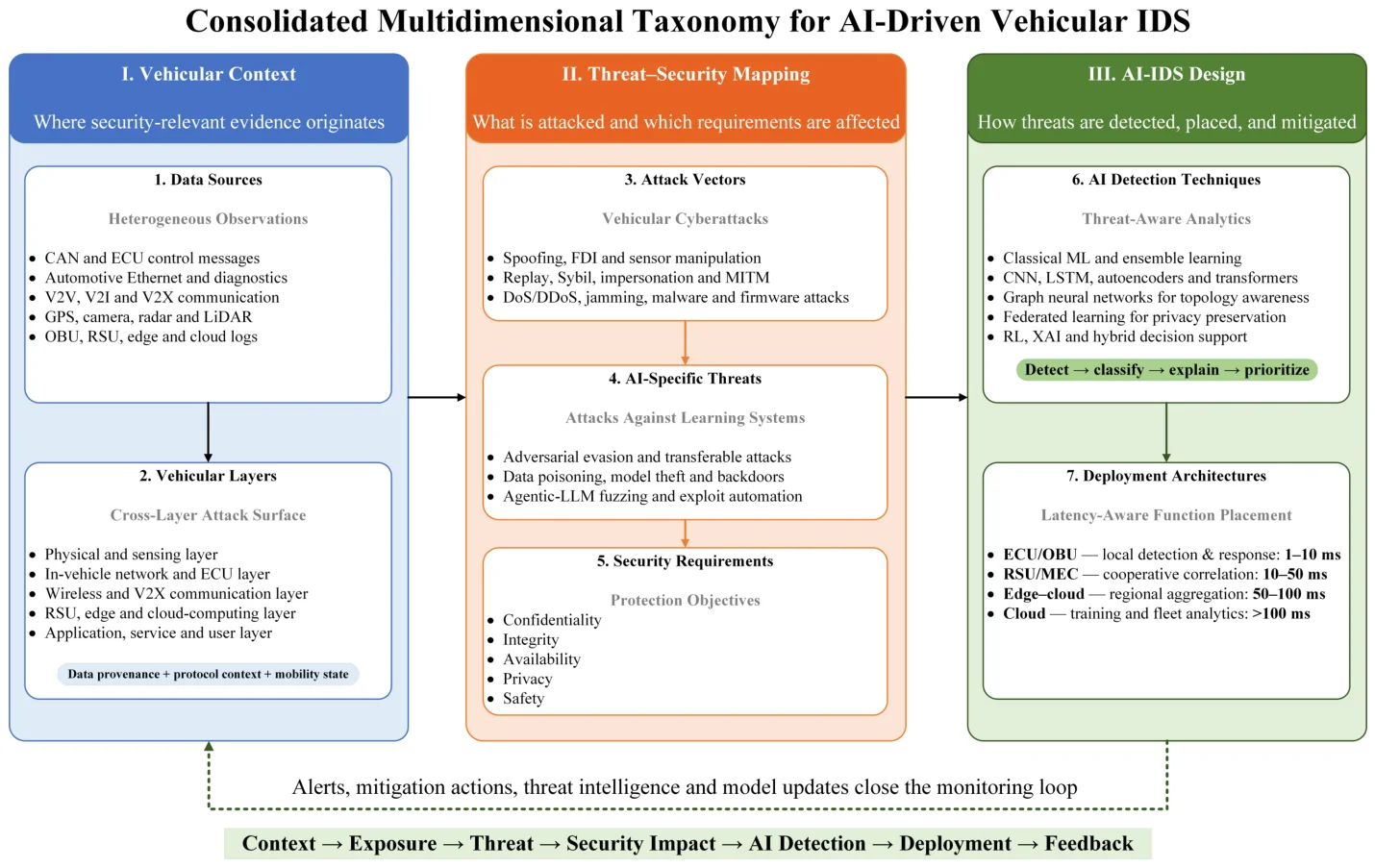
Multidimensional taxonomy linking vehicular data sources, system layers, attack vectors, security impacts, AI detection techniques, and deployment architectures.

For designing efficient intrusion detection systems in VANET, it is crucial to have a comprehensive taxonomy of attacks. Safwat et al. [136] demonstrated that vehicular attacks involve multiple communication layers and directly breach the three main rules: confidentiality, integrity, and availability (CIA). To further classify the attack taxonomy, Chen et al. [137] added attacks specific to IoV applications, e.g., data poisoning and model evasion. Rezazadeh Baee et al. [138] highlighted that authentication vulnerabilities pose a highly important problem, leading to identity-based attacks, while Chaubey and Yadav [139] noted that Sybil attacks are among the most serious threats in VANETs. Both Sri et al. [140] and Karim et al. [141] demonstrated that attacks can be leveraged across layers, impacting the reliability of the vehicular communication network. Dong et al. [142] and Verma et al. [143] showed that attacks on the system, such as replay, DDoS and false data injection, have a significant impact on system performance and Azzedin and Alhejri [144] stated that modern IoT-based vehicular systems include cross-layer and emerging attack vectors. Table 24 presents the multidimensional attack taxonomy for vehicular networks. presents the multidimensional threat taxonomy adopted in this survey. Figure 4 presents the multidimentional taxonomy linking vehicular data sources, system layers, attack vectors, security impacts, AI detection techniques, and deployment architectures. In summary, attacks mounted by vehicles result from several factors and necessitate an integrated IDS system that addresses multiple aspects of the network, applications, privacy, and AI-powered threats.

Cross-layer cascading attacks exploit the connectivity between external V2X interfaces and internal vehicle networks. An attack may begin through falsified V2X messages, a compromised RSU, cellular interface, or telematics service and subsequently target the OBU or central gateway. After compromising the gateway, the attacker can translate external messages into malicious CAN frames, enabling command injection, ECU impersonation, denial-of-service, or manipulation of safety-critical functions. The reverse path is also possible, where a compromised ECU uses V2X connectivity to exfiltrate data, spread malicious information, or coordinate attacks against nearby vehicles and infrastructure. Such attacks are difficult to detect using isolated CAN- or V2X-based IDSs because each monitor observes only part of the attack sequence. Effective protection therefore requires temporal correlation of CAN and V2X events, gateway-level access control, cross-layer feature fusion, device-identity verification, and coordinated detection across vehicles, RSUs, and edge servers. A multidimensional taxonomy for AI- Diven vehicular IDS is presented in Figure 4.

### 6.1. Network Layer Attacks

Sybil attacks are one of the most severe attacks that are possible in vehicular networks. Chaubey and Yadav [139] reported that attackers create numerous fake identities to generate fake traffic and make appropriate routing decisions. It is hard to detect because it resembles legitimate pseudonym changes. The network topology can be altered in a wormhole attack, as demonstrated by Karim et al. [141], and black holes can disrupt communication, as explained by Verma et al. [143]. Dong et al. [142] also proposed that La Grande Bear Hunger’s approach to reducing system availability is to flood network resources in DDoS attacks. The attacks exploit routing and communication protocols, so the system must include intelligent IDS techniques that can detect behavioural and topological anomalies. The network layer attacks and detection techniques are described in Table 25. In Table 24 and Table 25, the affected CIA properties column identifies the security properties that may be compromised by each attack: confidentiality (C), integrity (I), and availability (A). Confidentiality refers to unauthorized disclosure, inference, or tracking of vehicular information; integrity refers to unauthorized manipulation, injection, replay, or falsification of messages, identities, locations, or model updates; and availability refers to degradation, blocking, or disruption of communication and safety-critical services. The listed labels indicate the primary potential impact of an attack; an attack may affect more than one CIA property depending on its deployment context and severity.

### 6.2. Application Layer Attacks

Application-layer attacks aimed at vehicle networks directly target cooperative services or decision-making processes by injecting and manipulating higher-level communication messages. However, false data injection and spoofing attacks are possible and can compromise the integrity of sensing and control systems, potentially impacting vehicular decision-making processes, as described by Sun et al. [145]. Lopez et al. [146] presented an attack model for intelligent transportation systems and demonstrated how attacks such as falsified data injection or bogus warnings can greatly disrupt cooperative awareness services, leading to misguided or unsafe routing decisions. Tehrani et al. [147] studied system-level attacks on deep learning models in autonomous vehicles and showed that, with adversarial inputs or sensor input manipulation, application-level decision-making can be tampered with. App-layer attacks have an immediate effect on system-level functionality and service integrity; Abbas et al. [148] presented a multi-layer attack taxonomy that demonstrates this. Krishna et al. [149] analyzed IoT-based vehicular attacks and how they can compromise system integrity and disrupt reliable communication. Replay and API injection attacks emerged as challenging threats to system integrity and reliable communications. Likewise, Hassan et al. [150] investigated application-layer DoS/DDoS detection for vehicular networks using random forest classification and LIME-based explanations, demonstrating effective differentiation between benign and malicious traffic. Application-layer attacks pose a significant threat to vehicular systems, as they directly affect high-level services. This requires the development of intelligent, context-aware IDS tools to detect and address such attacks. The application layer attacks and detection techniques are described in Table 26.

### 6.3. Privacy Attacks

Most privacy attacks in vehicular networks exploit communication design and various identity management solutions, leading to the disclosure of users’ sensitive information. Ullah et al. [151] showed that there is a correlation between pseudonym changes across different observation points, which deconstructs vehicles’ trajectories and reveals sensitive location information. Authors in [152] demonstrated that identity spoofing attacks compromise vehicular authentication by exploiting vulnerabilities in cryptographic credentials, enabling spoofing of vehicles. Moreover, Gupta et al. [153] noted that in connected vehicle systems, such attacks would enable long-term vehicle tracking by associating multiple pseudonyms, thereby raising privacy concerns for users. These studies highlight the importance of advanced mechanisms for privacy and IDS protection in vehicular networks, particularly against privacy attacks based on identity management or communication transparency. Table 27 defines the privacy attacks in vehicular networks.

### 6.4. AI-Specific Attacks

Instead of attacking the communication infrastructure, AI-based attacks exploit the AI-based IDS model. Demonstrate that the predictions from a trained model can be manipulated by adversarial inputs and that the detection accuracy will suffer with these inputs by Pispa and Halunen [154]. Weebadu Arachchige [155] introduced a threat intelligence framework that noted that adversarial attacks can be ported across models to achieve black-box attacks. In adversarial machine learning (AML) attacks, Kiribuchi et al. [156] showed that attackers can exploit model decision boundaries to mislead the classifier into classifying malicious inputs as benign. Krawczyk et al. [157] demonstrated that data poisoning threats can affect training pipelines by corrupting samples and causing systematic failures in detection. The study in [158] also noted the severe security concerns posed by model evasion and adversarial manipulation, and Neupane et al. [159] showed that AI systems remain susceptible to gradient-based attacks and model-exploitation techniques. The results of this study show that AI-based IDS systems create a new attack surface that requires strong, adaptive defence strategies. Table 28 summarizes the AI-driven attacks on vehicular networks.

### 6.5. Agentic AI- and LLM-Enabled Attacks

The integration of large language models (LLMs) and agentic AI into vehicular systems creates new attack surfaces. Unlike conventional AI models, agentic systems can plan tasks, retain memory, communicate with other agents, access external tools, and execute actions with limited human supervision. Attackers may exploit these capabilities to automate reconnaissance, generate malicious CAN or V2X messages, identify vulnerabilities, coordinate multi-stage attacks, or adapt their strategies according to IDS responses.

These threats include prompt injection, jailbreaking, memory and knowledge-base poisoning, sensitive-data leakage, unsafe tool invocation, goal manipulation, and compromised inter-agent communication. In vehicular environments, malicious instructions may be introduced through infotainment systems, cloud services, traffic messages, maintenance records, or multimodal sensor inputs. A compromised agent could suppress security alerts, reveal private information, alter IDS rules, or initiate unauthorized actions. Detecting such attacks is challenging because malicious behaviour may be distributed across several apparently legitimate decisions. Therefore, vehicular IDSs should monitor prompts, memory updates, tool calls, and inter-agent messages while enforcing authentication, least-privilege access, input validation, runtime guardrails, and human approval for safety-critical actions.

Offensive LLM agents can strengthen vehicular attacks through autonomous payload synthesis, dynamic protocol fuzzing, and automated vulnerability exploitation. They may generate and repeatedly modify malicious CAN, automotive Ethernet, or V2X messages according to observed system responses, enabling attacks to evade fixed signatures. During protocol fuzzing, an agent can produce context-aware malformed messages, analyse resulting errors, and adapt subsequent inputs to discover weaknesses in protocol implementations. LLM agents may also analyse V2X firmware, configuration files, logs, and technical documentation to identify vulnerabilities and coordinate multi-stage exploitation with limited human involvement.

### 6.6. Additional Emerging Attacks in Vehicular Networks

Vehicular networks also face attacks targeting physical sensing, AI-based decision-making, and the automotive supply chain. Physical-layer and sensor-spoofing attacks manipulate GNSS, radar, LiDAR, camera, or wireless signals through jamming, replay, fabricated objects, and deceptive sensor inputs. These attacks can corrupt vehicle localization, environmental perception, and cooperative driving decisions while producing data that may appear legitimate to conventional IDSs.

Advanced adversarial AI and evasion attacks use carefully manipulated inputs to mislead AI-based intrusion detection and autonomous-driving models. Attackers may employ black-box queries, transferable adversarial examples, adaptive traffic modification, or model extraction to discover and bypass detection boundaries. Their continuously changing behaviour reduces the effectiveness of static classifiers and signature-based detection.

Supply-chain and ECU firmware attacks originate from compromised third-party software, hardware components, development tools, diagnostic interfaces, or over-the-air update mechanisms. Malicious or altered firmware may create persistent backdoors, disable security controls, manipulate ECU functions, or enable lateral movement across in-vehicle networks. Addressing these threats requires sensor-fusion validation, adversarially robust models, secure boot, signed firmware, software-component verification, and continuous integrity monitoring.

## 7. Performance Metrics and Comparative Evaluation

To systematically evaluate a vehicular IDS using AI, it is essential to select vehicle-specific, standardized metrics, define consistent experimental procedures, and account for vehicle operational limitations. This section provides a summary of the major evaluation metrics used in vehicular IDS research, presents a comparative performance evaluation of AI method categories, and concludes the discussion of the drawbacks of existing evaluation methodologies.

### 7.1. Evaluation Metrics

Evaluation of AI-based intrusion detection systems (IDSs) in the vehicular network is based on a set of classification and system-level metrics. In imbalanced vehicular datasets, normal traffic instances far outnumber attack instances, and detection accuracy (ACC) can be misleading, as shown by Moulahi et al. [160]. Hussain and Rani [161] demonstrated that precision and recall are more useful than other measures, with precision reflecting false alarm control and recall reflecting the system’s ability to detect real attacks. Additionally, Al Farsi et al. [162] noted that the F1 score balances precision and recall, which is appropriate for the IDS benchmark. Evaluating delays, computing overheads such as latency and memory consumption, and the false alarm rate (FAR) were recognized by Bouchouia et al. [163] as essential evaluation metrics for vehicular IDS, especially when deployed in real-time systems on resource-restricted OBUs. The results show that the important metrics for evaluation of IDS of road transport are both classification accuracy and operational efficiency metrics, and thus, these two aspects must also be considered for IDS deployment to be feasible. For OBU-deployed GNN and Transformer IDS models, predictive performance alone is insufficient to establish real-time feasibility. Evaluation should jointly report macro-F1, false alarm rate, parameter count, serialized model size, peak memory usage, FLOPs/MACs per inference window, processor utilization, energy per inference, throughput, and median and p95/p99 inference latency on clearly specified OBU hardware. These measurements should be examined under varying CAN/V2X message rates, graph sizes, sequence lengths, vehicle densities, and model-quantization settings. Training cost should be distinguished from inference cost, while communication overhead should be included when computation is offloaded to edge or cloud nodes. Such multi-objective evaluation enables Pareto-based comparison and may show that a compact model with slightly lower detection accuracy is more deployable than a highly accurate but resource-intensive model that violates memory, energy, or response-time constraints.

### 7.2. Performance Analysis and Discussion

A performance comparison of the above intrusion detection systems in vehicular networks clearly shows their advantages and disadvantages in terms of detection accuracy, computational complexity and deployment. The deep learning model, particularly the hybrid CNN–LSTM model, achieves the highest accuracy because it can model the spatial and temporal patterns of vehicular data. Next, there are some deep learning-based studies, such as [60], that have reported over 99% accuracy in complex attack detection, indicating better performance of these models. However, they need many computations and inference operations, which are not very convenient to implement on the limited resources of onboard units (OBUs). Further, long-range dependencies in communication patterns are modelled to achieve even more powerful detection capabilities in transformer-based models. However, such models perform well but require high memory and latency, limiting their practical use in real-time vehicle systems, as in [96].

An additional advantage of FL is the absence of the need to share distributed data. In [109], it is shown that the IDS based on FL is competitive in terms of accuracy and users’ privacy. However, needing constant communication between infrastructure and vehicles will incur overhead and may affect real-time performance. Reinforcement learning (RL) is a learning model that offers flexibility; it can learn from dynamic environments, which are well-suited to IDS approaches. As shown by studies such as [115], exploiting RL models can adapt their detection methods as attack patterns evolve. This will provide more flexibility but will also pose another challenge: increased training complexity and longer convergence time. Ensemble methods, on the other hand, are still considered viable solutions for real-world deployment, as classical machine learning is. As illustrated in [62], these techniques have high accuracy and low latency and are well-suited for the OBUs with limited computing power. Overall, the analysis found that there was no single right answer to all situations. These very accurate models require more computing power, whereas light models will not be as accurate as required. Further, there is no uniformity in the datasets or evaluation frameworks used across studies, which limits the fairness and replicability of their comparisons.

The reported results are not directly comparable because the studies use different datasets, attack classes, class distributions, validation protocols, and hardware. They also include both binary and multiclass classification tasks. Therefore, the reported values represent within-study findings only and are not used to rank model families universally. Table 29 presents a comparative analysis of AI techniques and Table 30 summarizes the performance trade-off between them.

## 8. Blockchain-Enhanced Vehicular IDS

This integration of blockchain with AI-powered vehicular IDS not only meets critical needs for trust, auditability, and tamper-resistance but also introduces new possibilities for secure data management and analysis. The decentralized nature of a distributed ledger within blockchain enables secure IDS alerts and logging, the sharing of threat data and intelligence in a decentralized way, and secure audit trails that meet regulatory requirements for reporting vehicular security events and cannot be altered.

Although blockchain improves the integrity and traceability of IDS alerts, consensus and ledger synchronization introduce additional delays. Existing blockchain-based vehicular IDS studies identify consensus, communication, and energy overheads [164,165,166,167], but their results cannot be directly compared because they use different consensus protocols, validator numbers, hardware platforms, and traffic loads. For consistent evaluation, end-to-end alert latency should be expressed as

(1) $$L_{\mathrm{alert}}=L_{\mathrm{detection}}+L_{\mathrm{transmission}}+L_{\mathrm{queue}}+L_{\mathrm{consensus}}+L_{\mathrm{commit}}.$$

The blockchain overhead can then be measured as the latency difference between blockchain and equivalent non-blockchain implementations. Benchmarking should vary alert-generation rate, vehicle density, validator count, payload size, and handover frequency while reporting median and p95/p99 finality latency, committed alerts per second, bandwidth per alert, CPU and memory utilization, energy consumption, and deadline-miss rate. Since the reviewed studies do not report all these measurements under common conditions, their overhead results remain descriptive rather than directly comparable. For safety-critical applications, immediate IDS decisions should therefore remain on the OBU or edge node, while blockchain recording and threat-intelligence sharing should be performed asynchronously.

### 8.1. Immutability and Audit Trail Capabilities

Attacks on infrastructure, crash investigations or legally contested incidents require a safe and secure way to capture the security alerts generated by a vehicular intrusion detection system (IDS). IDS frameworks on the blockchain provide an immutable audit trail: once an alert is recorded, it cannot be altered or deleted. Nakayaza et al. (2025) [168] proposed a data falsification detection model for 6G vehicular networks using multiple data logs (DLs) and data engineering and feature engineering techniques. They achieved good event-detection accuracy and dropped the requirement for storing IDS events, but adding high-frequency events to the stored events made storage overhead too high. Similarly, Aliyu et al. (2025) [169] proposed an IDS-based approach by combining blockchain and deep learning trusted execution environments. They were more reliable in determining their own reliability and used distributed ledgers to ensure secure logging, but their encryption/consensus incurred high computational costs. Furthermore, Nagarjun and Rajkumar (2025) [170] developed an IDS system using QDL technology, boasting Ethereum blockchain capabilities. Their approach enhanced fraud prevention and ensured the safety of data transfers, though it came with drawbacks, such as latency and computational complexity. Moreover, Gupta et al. (2024) [171] presented another secure third-party risk management approach using blockchain, applicable to vehicular IDS, but with scalability challenges in large, distributed setups. Table 31 presents the blockchain based IDS logging mechanisms.

### 8.2. Decentralized Threat Intelligence Sharing

However, centralized architectures with vehicular IDSs suffer from scalability issues and a single point of failure, especially when collecting sensitive data from vehicles. A decentralized system facilitated by blockchain technology notably enables the secure sharing of threat intelligence among actors in the vehicular ecosystem, without compromising data integrity or privacy. To address security challenges in EV infrastructure, Abid et al. [164] proposed a federated learning (FL)- and blockchain-based approach for anomaly detection (AD). The authors showed that their model could detect anomalies with higher accuracy without compromising data privacy, but noted a communication issue between distributed nodes. Huang et al. [165] proposed an IDS optimization scheme based on blockchain technology to enhance the detection efficiency by allowing secure neighboring nodes to share attack patterns. However, consensus took some time to impact real-time detection capacities.

The progress in vehicular networks suggests Firdaus et al. [166] for privacy-preserving vehicular communication with edge intelligence via blockchain. Though their system improved data-sharing efficiency and privacy protection, it also introduced additional latency in the blockchain synchronization process. Furthermore, Wang et al. [167] demonstrated a thorough understanding of blockchain intelligence for the Internet of Vehicles (IoV) and its usefulness for IoV decentralized threat intelligence sharing, while pointing out the trade-offs among energy consumption, security and latency. Table 32 presents the blockchain based vehicular IDS architectures.

## 9. Challenges and Open Issues

Nevertheless, despite the good work that has been done in the field of AI-based vehicular IDS (IDS), significant R&D challenges prevent the implementation of lab prototypes in real production IDS systems. This section provides a comprehensive analysis of the most critical challenges across the technical, operational, and regulatory aspects of the field. However, the availability of high-quality, real-world, labelled datasets is a fundamental limitation in the development of AI-based vehicular intrusion detection systems (IDSs). Due to safety, legal, and privacy considerations about vehicle communications, it has been difficult to gather data from vehicular attacks. Additionally, Sundfeldt et al. [172] discussed the difficulty of obtaining real vehicular attack datasets and noted that most IDS models are based on synthetic or simulated data, thereby limiting their applicability in the real world. Based on their results, they concluded that they are not seeing any real attack diversity, and the IDS’s generalization capability is poor. In a similar vein, Aljabri et al. [173] have presented an IDS system with lightweight characteristics for autonomous vehicles that detects attacks with high precision during testing; however, it is less accurate on out-of-test datasets due to the limited testing dataset. Tanaji and Roychowdhury [174] studied the limitations of datasets for connected vehicles and found the simulation [184-to-reality gap to be a significant problem, namely, that simulation models often fail to predict real-world vehicular dynamics. In addition, Jeyaram et al. [175] presented a real-time IDS system that highlighted the issues of class imbalance and missing attacks, which affect detection reliability.

A major challenge is detecting offensive LLM agents whose payloads, targets, and attack sequences continuously change. Existing datasets rarely include adaptive protocol fuzzing, AI-generated payloads, or autonomous V2X firmware exploitation. Future evaluation environments should therefore include realistic firmware, protocol state machines, and agent-generated attack traces. Effective protection requires state-aware intrusion detection, secure firmware validation, isolated testing environments, runtime monitoring of agent actions, and human authorization for safety-critical responses. Table 33 presents the key challenges in vehicular IDS.

### 9.1. Real-Time Detection Constraints

There is a strong constraint on the latency of vehicular IDS as it is a safety-critical application. In applications like collision avoidance and cooperative driving, detection decisions need to be made in just milliseconds so that one can respond in time and Robots can act accordingly. Aljabri et al. [176] introduced feature-engineered lightweight IDS models that significantly reduce inference time while maintaining competitive detection accuracy, making them suitable for on-board unit (OBU) deployment. He et al. [177] presented a hyper-dimensional computing method for real-time vehicular IDS that offers low-latency detection with minimal memory consumption but suffers from scalability issues. Although Kumar et al. [178] proposed a hybrid stacking model for a high-mobility vehicular environment, the detection accuracy was enhanced at the cost of high computational overhead. Further, authors in [179] investigated the trade-off between accuracy and efficiency of hybrid AI IDS and found that lightweight models can achieve near-optimal performance at the cost of reduced latency. Table 34 presents real-time constraints faced by vehicular IDSs.

### 9.2. Scalability in High-Mobility Environments

The network topology in vehicular networks is highly dynamic, posing challenges for IDS deployment, particularly in terms of scalability. IDS performance is degraded by frequent topology changes, high vehicle density and fast communication handovers. Laanaoui et al. [180] proposed a solution to the scalability problem using load balancing and observed certain limitations in the real-time processing of RSUs, but with improved scalability. El-Gayar et al. [181] developed a collaborative IDS and implemented it using ensemble learning to enable distributed detection of trespassers while enhancing scalability and detection capability. High-mobility environments are also discussed by Kumar et al. [178] using hybrid models, but issues with performance consistency across various traffic densities are noted. The scalability challenges faced by vehicular IDS are presented in Table 35.

### 9.3. Privacy and Security Trade-Offs

Vehicular IDS should rely on vehicle communication and behaviour to ensure detection, while balancing detection performance with the level of privacy maintained. Reddy et al. [182] presented an AI-powered, privacy-preserving framework for VANs that enables data sharing while preserving the accuracy of detection results. Basri [183] analysed privacy concerns with centralized data collection in VANETs and presented decentralized solutions, reviewing Vehicular Transport Networks (VANTs) and discussing different types of VANETs. AI-based communication system focused on secure V2V communication; however, the complex implementation is a constraint [184]. Table 36 presents the privacy trade-off in vehicular IDSs.

### 9.4. Adversarial Robustness

Vehicular networks’ AI-based IDS systems are susceptible to adversarial attacks in which malicious actors exploit input data to evade detection. Mansourian et al. [185] proposed adversarial-robust IDS models that are resistant to evasion attacks but consume additional computational power. Table 37 defines the open challenges in vehicular IDSs.

Han et al. [188] assessed adversarial robustness of IDSs (in adversarial settings) and showed that traditional ML-based models are very vulnerable to crafted adversarial attacks. Merzouk et al. [189] introduced an improved reinforcement learning-based robust IDS, but it was unable to operate in real time.

In federated IDSs, local-phase poisoning manipulates training samples, labels, or gradients at compromised vehicles, while evasion modifies runtime traffic features to bypass local detection. At the global aggregation phase, Byzantine, Sybil, colluding, or model-replacement updates can corrupt the shared model or implant backdoors. Local risks require input validation and adversarial training, whereas aggregation risks require update clipping, client verification, anomaly screening, and robust aggregation. Table 38 shows the key challenges in adversarial robustness in vehicular IDSs.

### 9.5. Standardization and Interoperability

There is a lack of standardized architecture of vehicular IDS, which poses an interoperability challenge between car producers and platforms. To enable integration across heterogeneous environments, Okoro [186] proposed an interoperability model for AI-based vehicular security systems. Authors in [187] highlighted the need for standardizing blockchain IDS in IoV, noting the lack of standards for IDS on blockchain.

## 10. Future Research Directions

**Figure 5:**
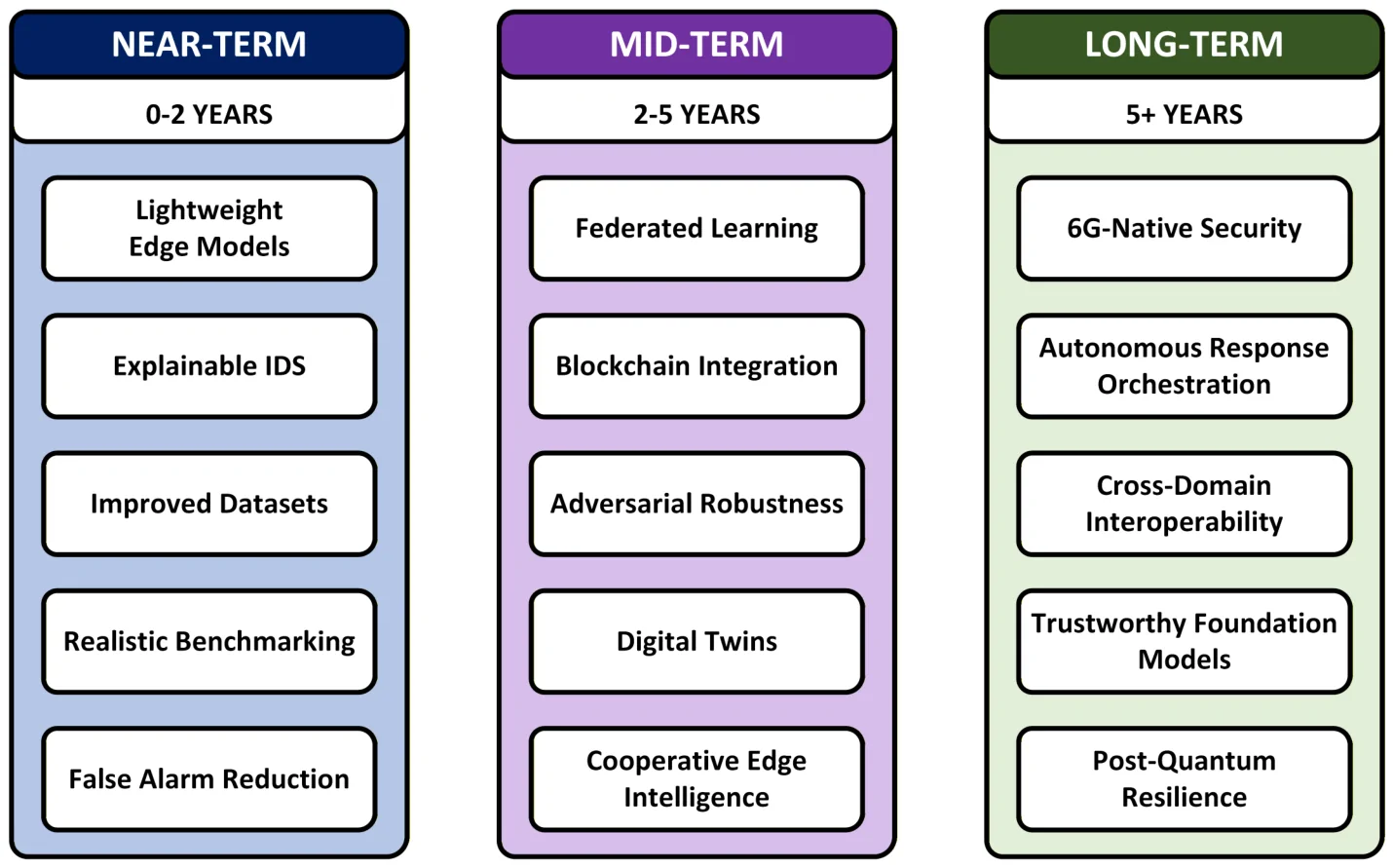
Research roadmap for next-generation AI-based vehicular IDS.

A list of high-priority research avenues has been identified based on existing shortcomings and outstanding challenges, guiding the next generation of AI-based vehicular IDS. This section summarizes some of the most exciting areas in which technology developments and gaps noted during this survey could be further developed to advance the state of the art. The research roadmap in Figure 5 summarizes near-, mid-, and long-term directions for next-generation vehicular IDS, including lightweight OBU models, XAI, adversarial robustness, FL-blockchain integration, digital twins, 6G edge-native IDS, post-quantum security, and interoperability.

The 6G vehicular networks will revolutionize intrusion detection systems (IDSs) with ultra-low delay, edge-based intelligence, and real-time, context-aware detection. Research recently revealed that incorporating AI at the network edge enables faster threat detection and greater scale. Multi-objective IDS models improve the accuracy in such environments; for instance, federated learning can reduce latency while maintaining privacy in V2X systems. Edge-centric architectures also help minimize response times by processing data locally. Nevertheless, there are challenges in the field, such as sparse edge resources and high communication overheads when deployed in a distributed system.

### 10.1. Explainable AI for Vehicular IDS

As deep learning-based IDS models are becoming more popular, concerns have arisen about their interpretability and transparency. A lack of transparency in decision-making processes in safety-critical vehicular systems will create difficulties for user trust, regulatory compliance, and forensic analysis. There has therefore been a critical need to incorporate Explanation as an important element in next-generation IDS design, a concept popularly known as Explanation-based Artificial Intelligence (XAI). To make this possible, Nwakanma et al. [29] introduced an XAI-based IDS that leverages model-agnostic explanation methods to enhance network interpretability without compromising detection capabilities. This was complemented by Lundberg et al. [31], who showed that feature attribution techniques greatly enhance the interpretability of IDS output, thereby aiding incident investigation. More recently, Taheri et al. [113] embedded XAI into federated learning architectures, enabling explainable federated ID. All of these methods introduce additional computational overhead and complexity to a system, especially in real-time systems.

### 10.2. Digital Twin-Based Security Frameworks

Digital twin technology has been used as a paradigm to boost the development and validation of vehicular IDS systems. Digital twins can be used to safely simulate attack scenarios on virtual copies of physical vehicular systems, detect anomalies in real time, and continuously monitor the system. The limitations of simulated environments were highlighted by Rajapaksha et al. [4], who stressed the potential of digital twins to provide realistic datasets for IDS training. Likewise, Almutairi [25] proposed a vehicular security framework based on simulation, demonstrating that the designed anomaly detection method achieves higher accuracy in certain experiments. Nevertheless, implementing digital twins requires high fidelity and synchronization, placing a heavy computational and infrastructural burden.

Digital-twin-driven IDSs require continuous synchronization between physical vehicles and their virtual replicas, creating substantial communication, storage, and computational overhead. Maintaining model fidelity under changing sensors, software, vehicle dynamics, and network conditions is also costly. Future research should investigate lightweight adaptive twins, event-triggered synchronization, and edge-assisted updates that balance detection accuracy with resource consumption.

### 10.3. Post-Quantum Cryptographic Integration

In the era of quantum computation, classical cryptographic protection systems will pose major problems for the transport of information between vehicles. However, evolving anomaly detection challenges in new environments, such as post-quantum cryptographic (PQC) environments, require adaptation of frameworks traditionally designed for classical computers and models. DS frameworks, as a result, need to be flexible to these new environments—post-quantum cryptographic (PQC)—where new communication patterns and computational overhead will affect how anomalies are detected. Hakeem and Kim [2] noted the importance of adapting IDS models to fit V2X systems running PQC and the need to incorporate cryptographic-aware behavioural profiling into future IDS. However, the inclusion of PQC introduces additional latency and computational complexity, requiring optimization of IDS architectures [135]. A comparative analysis of adversarial attacks and defense strategies in vehicular IDS are presented Table 39.

### 10.4. Adversarially Robust IDS Design

Attack resilience is a key concern of AI-based IDS models in vehicular networks. System reliability may be jeopardized as adversaries can potentially “manipulate” input data to avoid being detected. Aloraini et al. [33] show that the traditional IDS models are very susceptible to adversarial perturbations, thus necessitating proper defence mechanisms. In turn, Mansourian et al. [185] suggested that adversarially trained IDS models achieve higher evasion accuracy. Nevertheless, adversarial training also imposes an additional computational burden and may degrade real-time detection performance, especially in resource-limited mobile networks, such as those in vehicles.

### 10.5. Lightweight Models for Resource-Constrained OBUs

For use in onboard units (OBUs), lightweight, efficient models that meet strict resource constraints are required. Thus, optimizing the model is crucial to guarantee reliable detection accuracy in real-time applications. Aljabri et al. [176] proposed an IDS model that uses feature optimization techniques and results in less inference latency, even suitable for the deployment of OBU. Similarly, Yang et al. [59] proposed a hybrid IDS architecture with an efficient and effective detection system. Although these progressions have been made, finding an optimal trade-off between accuracy and computational efficiency remains a challenge.

### 10.6. Defense Against AI-Driven Attacks

Future vehicular IDS research should address attacks generated or enhanced by AI, including adaptive evasion, automated vulnerability discovery, intelligent malware, synthetic V2X messages, prompt injection, model poisoning, and coordinated agentic attacks. Such threats can continuously modify their behaviour to bypass static detection mechanisms. Future IDSs should therefore incorporate continual learning, adversarial training, multimodal monitoring, runtime agent supervision, and automated threat-intelligence sharing. Standardized datasets and realistic testbeds are also required to evaluate AI-generated attacks under mobility, latency, and resource constraints. Particular attention should be given to explainability, human oversight, and fail-safe mechanisms before autonomous defensive actions are deployed in safety-critical vehicular environments.

## 11. Conclusions

This survey is built to review and analyse the key aspects of artificial intelligence-based intrusion detection systems (AI-IDSs) for vehicular networks across various architectures, communication technologies, advanced techniques, datasets, and attack taxonomies for performance evaluation. It brings together several diverse research areas and thus offers a unified perspective on the newly established cybersecurity of the vehicular division. Several trends emerge from the results: vehicular networks are very complex and hostile cyber-physical systems; nodes exhibit very high mobility; communications are decentralized architectures have stringent latency requirements; and the system has limited resources. In such scenarios, conventional IDSs based on known-signature and rule-based approaches are inherently inappropriate, as they lack the capability to detect zero-day and adaptive attacks. In contrast, artificial intelligence-based approaches—such as deep learning, hybrid systems—as well as the new transformer-based systems can point to a much more powerful representation of complex space-time (S-T) patterns and be able to detect complex, multi-stage attacks. The comparison indicates that transformer-based and hybrid models (CNN-LSTM) deliver the best detection results. Moreover, in distributed vehicular ecosystems, federated learning is an important trade-off between privacy preservation and detection accuracy. Other advancements, such as reinforcement learning and multi-agent systems, further enhance the adaptability of IDS systems, enabling them to respond to threats in a context-aware, dynamic manner in non-stationary environments. A key finding of the survey is the limited availability of realistic vehicular datasets that integrate multiple communication layers, such as CAN, V2X, and SDV interfaces. This restricts the generalizability of current IDS models to real-world attack scenarios. Therefore, standardized cross-layer benchmark datasets are urgently needed for developing practical and robust vehicular IDS solutions. The proposed taxonomy links attack vectors with vehicle layers, CIA requirements, and AI-specific threats. Emerging adversarial attacks highlight the need for robust, explainable, and attack-resistant IDS frameworks.

## Figures and Tables

**Table 1:** Comparative analysis with existing review studies.

Study Characteristics	[1]	[2]	[3]	[4]	[5]	[6]	[7]	[8]	Our Work
Vehicular-specific focus	×	✓	✓	✓	×	✓	✓	✓	✓
AI taxonomy provided	✓	✓	✓	✓	✓	∼	✓	✓	✓
Dataset analysis	✓	∼	✓	∼	∼	∼	×	∼	✓
FL integration	×	✓	✓	×	×	×	×	×	✓
Adversarial attacks	∼	✓	✓	×	×	×	×	×	✓
XAI coverage	∼	∼	∼	×	×	×	×	×	✓
Blockchain-IDS	×	∼	×	×	×	∼	×	×	✓
Performance metrics	✓	✓	✓	✓	✓	✓	∼	✓	✓
5G/6G integration	×	✓	∼	×	×	∼	✓	∼	✓
Real-time deployment	∼	✓	✓	∼	×	✓	✓	∼	✓
Open challenges	✓	✓	✓	✓	✓	∼	✓	✓	✓

Note: ✓ = comprehensive coverage, ∼ = partial coverage, × = not covered.

**Table 2:** Vehicular network architecture studies and IDS relevance.

Ref.	Methodology	Architecture Focus	Technique	Key Result	IDS Contribution
[10]	AI-based IDS for V2V communication	VANET (OBU communication)	ML-based anomaly detection	High detection accuracy in dynamic V2V traffic	Enables real-time anomaly detection
[11]	Cellular-based V2V and V2I communication model	IoV (Cellular V2X)	Network-based modelling	Improved connectivity and reliability	Identifies vulnerabilities in cellular vehicular networks
[12]	V2P communication architecture analysis	Pedestrian–vehicle interaction	System-level modelling	Enhanced safety communication	Highlights new attack surfaces in V2P systems
[13]	Comprehensive V2X architecture overview	VANET + IoV integration	Conceptual framework	Unified V2X communication model	Defines a multi-layer attack surface for IDS design
[14]	SDN-based V2X architecture	5G-enabled V2X	SDN + network optimization	Improved network efficiency and flexibility	Exposes control-plane vulnerabilities requiring IDS monitoring

**Table 3:** V2X communication technologies based on existing studies.

Ref.	Technology	Methodology	Standard/Interface	Key Result	Security/IDS Implication
[15]	C-V2X	Cellular communication framework	3GPP-based architecture	Improved coverage and scalability over DSRC	Expands the attack surface in cellular vehicular networks
[16]	Advanced C-V2X	Standard evolution analysis	3GPP Rel. 14+	Supports URLLC for autonomous driving	Vulnerable to interference and spectrum attacks
[17]	5G-V2X	System architecture + use-case analysis	5G NR-V2X	Sub-ms latency and massive connectivity	Edge computing introduces distributed attack vectors
[18]	5G-V2X Optimization	Performance and challenge analysis	5G communication model	Improved efficiency with adaptive strategies	Security risks in dynamic network conditions
[19]	LTE-V2X/5G-V2X	Resource allocation modelling	Cellular V2X	Enhanced spectrum efficiency	Susceptible to resource manipulation and DoS
[20]	5G/6G V2X	Cyber threat intelligence framework	Next-gen V2X	Future-ready intelligent communication systems	Requires AI-driven IDS for large-scale threats

**Table 4:** Vehicular network security requirements and IDS implications.

Ref.	Security Focus	Methodology	Key Issue Addressed	Key Result	IDS Contribution
[21]	Secure communication in vehicular networks	Simulation and validation framework	Reliable data exchange in dynamic environments	Identifies need for robust communication security	Supports IDS design for dynamic vehicular networks
[22]	CAM-based communication security	Network performance analysis	Integrity and replay attacks in CAM messages	Highlights vulnerability in cooperative messaging	Enables IDS for message validation and anomaly detection
[23]	Sensor and safety communication security	System-level security analysis	Integrity and availability in emergency systems	Safety-critical systems highly attack-sensitive	Guides IDS for sensor data protection
[24]	Authentication and freshness	Safety application analysis	Replay attacks and outdated message risks	Importance of timestamp and validation mechanisms	Supports IDS for temporal anomaly detection
[25]	Confidentiality and privacy	IoT-based vehicular framework	Data leakage and tracking risks	Identifies privacy challenges in IoV	Enables IDS for privacy-preserving detection
[26]	Trust and privacy in VANETs	Survey and framework analysis	Identity spoofing and tracking	Need for decentralized trust mechanisms	Supports IDS for trust and identity anomaly detection

**Table 5:** Threat models and adversarial capabilities in vehicular networks.

Ref.	Threat Type	Methodology	Attack Scenario	Key Result	IDS Implication
[28]	External attacker	AI-based IDS framework	Eavesdropping, jamming, message injection	Identifies communication-layer vulnerabilities	Supports IDS for wireless attack detection
[29]	AI-based adversary	XAI-based security analysis	Model evasion and explainability exploitation	AI models vulnerable to intelligent attacks	Requires an explainable and robust IDS
[30]	Internal attacker	AI-based security framework	False data injection, routing manipulation	Compromised vehicles disrupt network operations	Enables IDS for insider threat detection
[31]	Insider (ECU/vehicle)	Experimental IDS evaluation	Malicious ECU behaviour, rogue nodes	Insider threats difficult to detect	Supports IDS for in-vehicle anomaly detection
[32]	Coordinated attack	ML-based cybersecurity framework	Sybil and distributed DDoS attacks	Multi-node attacks increase detection complexity	Requires cooperative and distributed IDS
[33]	Adversarial ML attack	Adversarial attack modelling	Model evasion and data poisoning	AI-based IDS vulnerable to adversarial inputs	Necessitates robust and adversarial-aware IDS

**Table 6:** IDS objectives and performance requirements in vehicular networks.

Ref.	Methodology	IDS Objective	Key Technique	Key Result	IDS Contribution
[34]	Survey and meta-taxonomy	Real-time IDS in vehicles	System-level analysis	Identifies constraints of vehicular IDS	Defines requirements for low-latency IDS
[35]	Deep learning-based IDS	High detection accuracy	DL feature extraction	Improved performance on CAN/V2X data	Enhances detection capability in vehicular traffic
[36]	Comprehensive IDS review	Reliability and robustness	Analytical evaluation	Highlights false positives and imbalance issues	Guides IDS design for real-world scenarios
[37]	Optimization-based IDS	Low latency and high accuracy	Many-objective optimization	Achieves efficient and accurate detection	Suitable for safety-critical vehicular systems
[38]	Transfer learning IDS	Efficient training and detection	Deep transfer learning	Reduces training time, improves accuracy	Enables scalable IDS deployment
[39]	Intelligent IDS framework	Adaptive and real-time detection	AI-based adaptive model	Improves real-time detection efficiency	Supports autonomous vehicle security

**Table 7:** Signature-based IDS approaches in vehicular and IoT environments.

Ref.	Methodology	Algorithm/Technique	Key Result	Use Case	Limitation
[40,41]	Signature-based IDS for CAN bus	Pattern matching (message ID)	High accuracy for known attacks	In-vehicle CAN network security	Cannot detect unknown attacks
[41]	Hybrid IDS (signature + anomaly)	Signature matching + anomaly detection	Improved detection performance	IoT and V2G communication	Requires hybrid integration
[42]	Signature-based IDS for IoT	Rule-based pattern matching	Low computational overhead	Resource-constrained systems	High maintenance and scalability issues
[43]	Combined IDS framework	Signature + behaviour-based detection	Enhanced detection accuracy	Intelligent transportation systems	Increased system complexity
[44]	Secure signature-based IDS	Pattern-based detection model	High accuracy in smart transport	Vehicular communication security	Limited adaptability to new attacks
[45]	Hybrid IDS component analysis	Signature-based module evaluation	Efficient detection for known threats	Hybrid IDS systems	Ineffective for zero-day attacks

**Table 8:** Anomaly-based IDS approaches in vehicular networks.

Ref.	Methodology	Technique	Key Result	Dataset	Limitation
[46]	Anomaly-based IDS for in-vehicle networks	Statistical anomaly detection	Effective detection of unknown attacks	Vehicular data	Sensitive to behaviour variation
[47]	ML-based IDS for VANET	Classification-based anomaly detection	Enhanced detection accuracy	VANET/IIoT data	Depends on the quality of training data
[48]	Deep SVDD-based IDS	Deep anomaly detection	Accurate detection in dynamic networks	Vehicular dataset	Computational complexity
[49]	Performance evaluation study	Comparative anomaly methods	Moderate accuracy with higher FAR	VANET datasets	High false alarms
[50]	GNN-based IDS	Graph neural networks	Robust detection performance	In-vehicle network data	Model complexity
[51]	Survey of anomaly-based IDS	ML/DL-based models	Improved detection with advanced models	Multiple datasets	High false positive rates

**Table 9:** Specification-based IDS approaches in vehicular networks.

Ref.	Methodology	Technique	Key Result	Use Case
[52]	Specification-based IDS for CPS	Rule-based behaviour modelling	Low false positives	Cyber-physical systems
[53]	Protocol-based IDS	Formal specification rules	Accurate protocol violation detection	Industrial/vehicular systems
[54]	Hybrid IDS (spec + AI)	Rule-based + ML methods	Improved detection performance	Modern vehicular networks
[55]	Vehicular IDS framework	Specification-based detection	Suitable for defined protocols	In-vehicle systems
[56]	Graph-based IDS	Graph modelling of communication	High detection accuracy	CAN protocol security
[57]	Pattern-based IDS	Generic specification patterns	Flexible IDS deployment	Automotive architectures

**Table 10:** Hybrid IDS approaches in vehicular networks.

Ref.	Methodology	Technique	Key Result	Limitation
[58]	Hybrid IDS (signature + anomaly)	Rule-based + ML detection	Improved detection accuracy	Increased system complexity
[59]	Multi-tier hybrid IDS	Layered IDS architecture	Efficient multi-level detection	Communication overhead
[60]	Deep learning hybrid IDS	CNN + LSTM	High accuracy (spatio-temporal features)	Computationally intensive
[61]	Hybrid DL with feature selection	Optimized deep learning	Improved accuracy and efficiency	Model tuning required
[62]	Hybrid ML ensemble	Multiple ML classifiers	Robust detection performance	Increased computation
[63]	Hybrid ML-based IDS	Combined ML models	Better performance than single models	High processing cost
[64]	Hybrid deep learning IDS	Multi-layer DL architecture	High accuracy in IoV	Resource-intensive

**Table 11:** IDS deployment architectures in vehicular networks.

Ref.	Methodology	Architecture	Key Result	Advantage	Limitation
[65]	Centralized IDS	Cloud-based aggregator	Effective large-scale attack detection	High computational capability	High latency and communication overhead
[66]	Distributed IDS (Auto-CIDS)	Vehicle-level detection	Fast local threat detection	Low latency, decentralized	Limited OBU resources
[67]	DL-based IDS	In-vehicle deployment	Real-time detection using CNN	Low latency	Resource constraints
[68]	Edge-based IDS	Automotive Ethernet/edge nodes	Improved scalability and efficiency	Reduced communication delay	Coordination complexity
[69]	Multi-stage IDS	Hierarchical (vehicle + cloud)	High detection accuracy	Scalable and flexible	Increased system complexity
[70]	Hybrid/Federated IDS	Distributed + cloud integration	Balanced privacy and performance	Privacy-preserving detection	Communication overhead
[71]	Real-time ML IDS	Edge-based deployment	Predictive threat detection	Real-time response	Resource management challenges

**Table 12:** Supervised machine learning approaches in IDS.

Ref.	Methodology	Algorithm	Key Result	Dataset	Limitation
[72]	Supervised IDS for IoT	Deep supervised learning	High detection accuracy	IoT dataset	Requires labelled data
[73]	ML-based IDS	RF, SVM	Accurate and efficient detection	Network datasets	Limited generalization
[74]	Supervised ML IDS	Decision Tree, RF	High accuracy in attack detection	System data	Depends on feature quality
[75]	Hybrid ML IDS	Supervised + unsupervised	Improved detection performance	Mixed datasets	Increased complexity
[76]	Adversarial analysis	Supervised ML models	Vulnerable to adversarial attacks	Network datasets	Security weaknesses
[77]	IoT IDS	Supervised ML models	Effective for known attacks	IoT data	Poor performance on unseen attacks

**Table 13:** Unsupervised learning approaches in IDS.

Ref.	Methodology	Algorithm	Key Result	Limitation
[78]	Unsupervised IDS for WSN	Clustering-based detection	Detects anomalous traffic patterns	Sensitive to noise
[79]	ICS anomaly detection	Statistical + clustering methods	Effective behaviour deviation detection	Depends on normal profile accuracy
[80]	Review of IoT IDS	Unsupervised ML models	Strong zero-day detection capability	High false positive rate
[81]	Hybrid anomaly detection	Multiple ML techniques	Improved detection accuracy	Increased complexity
[82]	Network anomaly detection	Unsupervised ML model	Identifies traffic anomalies	Limited precision
[83]	Federated IDS	Unsupervised + supervised DL	Scalable and adaptive detection	Communication overhead

**Table 14:** Deep learning (CNN-based) IDS approaches.

Ref.	Methodology	Model	Key Result	Dataset	Limitation
[84]	CNN-based IDS	1D-CNN	Accurate detection from raw data	Network traffic data	Requires training data
[85]	CNN anomaly detection	CNN model	Improved anomaly detection accuracy	Network dataset	Resource intensive
[86]	DL-based IDS review	CNN and DL models	Outperforms classical ML	Multiple datasets	Computational cost
[87]	Hybrid deep learning IDS	CNN-based hybrid model	High detection accuracy	Network dataset	Increased complexity
[88]	Hybrid CNN-RNN IDS	CNN + RNN	Detects sequential attacks effectively	Network dataset	High computation
[89]	RNN-based IDS	Recurrent neural network	Captures temporal dependencies	Network data	Slow training

**Table 15:** LSTM and recurrent IDS approaches.

Ref.	Methodology	Model	Key Result	Dataset	Limitation
[90]	RNN-based IDS evaluation	LSTM vs RNN	LSTM outperforms traditional RNN	Network datasets	Higher complexity
[91]	Optimized LSTM IDS	LSTM	High detection accuracy	Network dataset	Requires tuning
[92]	DL comparative study	LSTM, DNN, RNN	LSTM performs best for sequential attacks	Network data	Training overhead
[93]	BiLSTM IDS	Bidirectional LSTM	Improved accuracy using temporal context	Network dataset	Increased computation
[94]	Hybrid DL IDS	LSTM + feature extraction	Enhanced spatial-temporal detection	Network dataset	Complex architecture
[95]	Ensemble DL IDS	Recurrent ensemble model	Improved robustness and accuracy	Network data	High computational cost

**Table 16:** Hybrid CNN-LSTM and transformer-based IDS approaches.

Ref.	Methodology	Model	Key Result	Dataset	Limitation
[96]	Hybrid DL IDS	CNN + LSTM + Transformer	High detection accuracy	Network dataset	High computational cost
[97]	Hybrid IDS framework	CNN–LSTM–Transformer	Improved anomaly detection	Network data	Complex architecture
[98]	Spatial-temporal IDS	CNN + LSTM	Robust feature extraction	IoT dataset	Resource intensive
[99]	Hybrid CNN- transformer IDS	CNN + Transformer	Better long-range dependency modelling	Network dataset	Training complexity
[100]	Transformer-based IDS	CNN + Transformer	High accuracy in IoT IDS	IoT data	Requires large data
[101]	Explainable hybrid IDS	CNN + Transformer + XAI	Improved interpretability and accuracy	IoT/IIoT data	Increased overhead
[102]	Transfer learning IDS	Transformer + LSTM	High performance via transfer learning	IoT dataset	High computation

**Table 21:** Traditional network security datasets for vehicular IDS applicability.

Ref.	Dataset	Key Features	Key Result	Applicability	Limitation
[126]	KDD/NSL-KDD	Legacy network traffic	Useful for benchmarking	Very low for vehicular IDS	Outdated, no V2X data
[127]	Traditional IDS datasets	General network features	Limited relevance to vehicles	Low applicability	No vehicular protocols
[128]	ROAD (automotive dataset comparison)	CAN-based traffic	Reveals performance gaps	Medium (automotive-specific)	Limited dataset size
[129]	AI attack datasets	Modern attack patterns	Highlights evolving threats	Medium	Lacks vehicular context
[130]	CICIDS datasets	Diverse modern attacks	Improved detection evaluation	Medium	No mobility/V2X features

**Table 22:** Quantitative comparison of vehicular-specific intrusion-detection datasets.

Dataset	Data Origin	Quantitative Scale	Attack Classes	Data Modalities	Class Distribution	Source	Main Limitation
HCRL Car-Hacking (2018)	Real-vehicle CAN traffic captured through OBD-II	Four attack captures, each 30–40 min; 300 attack intervals per capture; each interval 3–5 s; separate normal capture	Normal, DoS, fuzzing, gear spoofing and RPM spoofing	Timestamp, CAN ID, DLC, eight payload bytes and binary injection flag	Normal and injected frames are identified, but the ratio differs across attack files	https://ocslab.hksecurity.net/Datasets/car-hacking-dataset (accessed on 25 September 2026)	Single vehicle; limited to CAN and relatively simple message-injection attacks
CAN-Intrusion (2017)	Real Kia Soul CAN traffic collected through OBD-II	Four released conditions; normal plus three attack scenarios; exact duration is not reported on the official page	Normal, DoS, fuzzing and impersonation	Timestamp, CAN ID, DLC and eight payload bytes	DoS frames can be identified by ID; fuzzing and impersonation lack complete per-frame labels	https://ocslab.hksecurity.net/Dataset/CAN-intrusion-dataset (accessed on 25 September 2026)	Coarse or incomplete labels for some attacks; single vehicle
SynCAN (2020)	Fully synthetic CAN signal-space data	Four training archives and six test sets; 10 CAN IDs containing 20 signals	Normal, plateau, continuous drift, playback, suppression and flooding	Label, CAN ID, timestamp and up to four signal values per ID	Training data are entirely benign; attacks occur only in designated test intervals	https://github.com/etas/SynCAN (accessed on 25 September 2026)	No physical vehicle, ECU or environmental variability
ROAD (2020/2024 archive)	Real CAN data collected from one vehicle using an automotive dynamometer	More than 3.5 h of ambient and attack traffic	Real fuzzing, fabrication and targeted attacks; simulated masquerade attacks	Raw CAN frames and signal-translated time series	Capture-specific; no standardized global class ratio	https://zenodo.org/records/10462796 (accessed on 25 September 2026)	One vehicle; dynamometer environment; some advanced attacks are simulated
VeReMi (2018)	Simulated VANET using LuST and VEINS; GPS and DSRC Basic Safety Messages	3.6 GB; three traffic densities × three attacker densities × five attack types	Constant position, constant offset, random position, random offset and eventual-stop attacks	Per-vehicle GPS, BSM reception logs and message-level ground truth	Controlled attacker-density settings rather than naturally occurring prevalence	https://github.com/VeReMi-dataset/VeReMi-dataset.github.io/blob/main/veremi.md (accessed on 25 September 2026)	Simulated mobility and wireless channel; simple attacks; no CAN traffic
VeReMi Extension (2020)	Simulated urban and highway V2X traffic using F2MD, VEINS and SUMO	Approximately 1.61 km^2^; 07:00–09:00 and 14:00–16:00 subsets plus a 24-h mixed set; 30% attackers	18 listed variants covering position, speed, delay, replay, DoS, Sybil and eventual-stop behaviours	V2X messages, mobility variables, sensor-error models, pseudonym changes and ground truth	Fixed 30% attacker penetration, which is higher than expected operational prevalence	https://github.com/VeReMi-dataset/VeReMi-dataset.github.io/blob/main/veremi-extension.md (accessed on 25 September 2026)	Simulation-only; no real radio traces or in-vehicle CAN data
can-train-and-test (2023/2024)	Real CAN traffic from four vehicles made by Chevrolet and Subaru	Four vehicles, two manufacturers and nine attacks applied to every vehicle, giving 36 vehicle–attack combinations	Nine attacks, including DoS, fuzzing, gear spoofing and standstill manipulation	Replayable CAN logs and labelled/unlabelled CSV files	No single global ratio because data are organized into multiple training and testing subsets	https://data.dtu.dk/articles/dataset/can-train-and-test/24805533 (accessed on 25 September 2026)	CAN-only; controlled attacks and no V2X or sensor modalities
CAN-MIRGU (2024)	CAN traffic from a real moving vehicle with autonomous-driving functions	17 h benign data; 2 h 54 min attack data; 26 physically verified attacks targeting 13 CAN IDs plus 10 simulated attacks; 497.1 MB archive	DoS, fuzzing, replay, spoofing, suspension and masquerade	Raw CAN logs and structured attack metadata	Benign traffic dominates by duration: approximately 85.4% benign versus 14.6% attack data	https://archive.ics.uci.edu/dataset/1035/can-mirgu (accessed on 25 September 2026)	Single vehicle; suspension and masquerade scenarios are simulated; CAN-only
CICIoV2024 (2024)	Real CAN traffic from the intact ECUs of a stationary, disassembled 2019 Ford vehicle	1,408,219 records: 1,223,737 benign; 74,663 DoS; 54,900 RPM; 24,951 speed; 19,977 steering; and 9991 gas-spoofing records	Benign, DoS, RPM spoofing, speed spoofing, steering-wheel spoofing and gas spoofing	CAN ID, eight payload bytes and three label fields; hexadecimal, decimal and binary versions	Strongly imbalanced: 86.9% benign; individual attack classes represent 0.7–5.3%	https://www.unb.ca/cic/datasets/iov-dataset-2024.html (accessed on 25 September 2026)	Stationary single-vehicle experiment; substantial repeated frames; no mobility or V2X traffic
Context-Aware VCN Dataset (2025)	Simulated vehicular communication with mobility and spatiotemporal attack modelling	120,602 instances: spoofing 46,564; wormhole 57,456; Sybil 16,582	Position spoofing, wormhole and Sybil attacks	Mobility dynamics, vehicle positions, inter-vehicle distances and end-to-end communication features	Highly imbalanced: malicious ratios are 1.26% for spoofing, 9.08% for wormhole and 10.57% for Sybil; 6.27% overall	https://www.tandfonline.com/doi/full/10.1080/08839514.2025.2538453 (accessed on 25 September 2026)	Simulation-only; only three attacks; no verified standalone repository URL
PQ-V2X (2025)	Hybrid V2X dataset combining cryptographic traces, mobility and zone-aware channel conditions	Five million labelled records; 23 attack types; 10.05% real PQC traces	Classical, hybrid and post-quantum attacks involving Kyber, Dilithium, Falcon and SPHINCS+	Cryptographic artifacts, mobility, channel-zone and resource-consumption features	Full per-class distribution is not reported in the publicly visible abstract	https://ieeexplore.ieee.org/document/11193862/ (accessed on 25 September 2026)	Mostly synthetic/hybrid records; stable standalone repository URL should be stated explicitly

**Table 23:** Critical research gaps in vehicular IDS datasets.

Ref.	Research Gap	Description	Impact on IDS	Proposed Solution
[131]	Simulation–Reality Gap	Simulation datasets lack real-world variability	Poor model generalization	Hardware-in-the-loop and real-world data collection
[133]	Class Imbalance	Limited attack samples vs. normal traffic	Biased detection performance	SMOTE, GAN-based augmentation
[127]	Emerging Attack Coverage	Lack of adversarial, SDV, and PQ attacks	IDS not robust to new threats	Include adversarial and OTA attack data
[126]	Protocol Coverage	Absence of CAN + V2X unified datasets	Incomplete IDS evaluation	Multi-layer integrated datasets
[127]	Single-Interface Focus	Isolated dataset design (CAN or V2X only)	No end-to-end security evaluation	Cross-layer dataset development

**Table 24:** Multidimensional attack taxonomy in vehicular networks.

Attack Category	Attack Type	Affected CIA Properties (C/I/A)	Target Layer	Detection Difficulty	Reference
Network Layer	General Vehicular Attacks	C, I, A	Network	High	[136]
AI-Specific	Data Poisoning	I	Training	Very High	[137]
Privacy	Identity Spoofing	C, I	Cross-layer	High	[138]
Network Layer	Sybil Attack	C, I	Network	High	[139]
Application	False Data Injection	I	Application	High	[140]
Network Layer	Wormhole Attack	C, I, A	Network	High	[141]
Application	Replay Attack	C, I	Application	Medium	[142]
Network Layer	Blackhole Attack	A	Network	Medium	[143]
Privacy	Location Tracking	C	Cross-layer	Low	[144]

Note: C = confidentiality; I = integrity; A = availability.

**Table 25:** Network layer attacks and detection techniques.

Attack	Mechanism	Affected CIA Properties (C/I/A)	AI Detection Method	Reference
Sybil Attack	Multiple fake identities	C, I	Graph-based detection	[139]
GPS Spoofing	Fake location signals	I	Position validation	[140]
Wormhole Attack	Tunnel-based topology attack	C, I, A	Latency/topology analysis	[141]
DDoS/Flooding	Traffic flooding	A	ML-based classification	[142]
Replay Attack	Message reuse	C, I	Timestamp validation	[142]
Blackhole Attack	Packet dropping	A	Traffic anomaly detection	[143]

Note: C = confidentiality; I = integrity; A = availability.

**Table 26:** Application-layer attacks and detection approaches.

Ref.	Attack Type	Methodology	Target Component	Key Result	Limitation
[145]	False data injection/spoofing	Attack classification in IVC systems	In-vehicle components	Identifies critical attack vectors	Limited real-time validation
[146]	False data/bogus warning	ITS attack modelling framework	Traffic management systems	Demonstrates impact on routing	Limited real-world evaluation
[147]	Adversarial input attacks	Deep learning attack modelling	Autonomous vehicle models	Detects model manipulation risks	High computational complexity
[148]	Multi-layer application attacks	IoT-based taxonomy modelling	Vehicular IoT systems	Structured attack categorization	Generalized framework
[149]	Replay/API injection	IoT threat analysis	Vehicular communication systems	Highlights system vulnerabilities	Lack of dedicated datasets
[150]	Application-layer DoS/DDoS	Random forest with LIME	VANET intrusion detection	Accurate, interpretable traffic classification	Robustness to changing vehicular traffic remains unverified

**Table 27:** Privacy attacks in vehicular networks.

Ref.	Attack Type	Methodology	Target	Key Result	Limitation
[151]	Location Tracking	Pseudonym correlation analysis	Vehicle identity	Reconstructs movement patterns	Requires multiple observation points
[152]	Identity Spoofing	Credential exploitation	Authentication system	Enables vehicle impersonation	Depends on PKI weaknesses
[153]	Linkability Attack	Identity correlation techniques	Communication layer	Enables long-term tracking	Hard to prevent without strong anonymization

**Table 28:** AI-specific attacks on vehicular IDS.

Ref.	Attack Type	Methodology	Target	Key Result	Limitation
[154]	Adversarial ML	Input perturbation attacks	ML models	Causes misclassification	Requires model sensitivity
[155]	Transferable attacks	Surrogate model attack	Black-box IDS	Enables cross-model evasion	Needs attack modelling
[156]	Model evasion	Decision boundary exploitation	Classifier	Bypasses IDS detection	Complex crafting process
[157]	Data Poisoning	Malicious training data injection	Training pipeline	Degrades model performance	Hard to detect
[158]	Adversarial manipulation	Real-time attack generation	AI systems	Affects system decisions	High computational cost
[159]	Gradient-based attack	Model exploitation	Deep learning models	Extracts model vulnerabilities	Requires model access

**Table 29:** Comparative analysis of AI technique categories for vehicular IDS by performance and trade-offs.

Ref.	AI Category	Representative Model	Strength	Primary Limitation
[28]	Classical Machine Learning	Random Forest	Lightweight and low-latency detection	Limited zero-day attack adaptability
[60]	Deep Learning	CNN–LSTM Hybrid	Excellent spatial–temporal feature learning	High computational overhead
[62]	Ensemble Learning	RF + SVM + DT Ensemble	Robust classification stability	Redundant computation and increased complexity
[96]	Transformer-Based IDS	CNN–LSTM–Transformer	Superior long-range dependency modelling	High inference latency and memory consumption
[103]	Graph Neural Networks	Temporal GNN	Effective topology-aware attack detection	Scalability and training complexity
[109]	Federated Learning IDS	FedAvg-CNN	Privacy-preserving collaborative learning	Communication overhead
[115]	Reinforcement Learning IDS	Multi-Agent RL	Adaptive intrusion response capability	Slow convergence and training instability
[121]	Generative AI IDS	GAN	Effective zero-day and anomaly detection	GAN instability and high training cost

**Table 30:** AI-IDS performance trade-off summary.

Ref.	AI Technique	Inference Latency	Resource Requirement	Privacy Preservation	Zero-Day Detection Capability	OBU Deployability	Scalability	Main Trade-Off
[62]	RF + SVM	Very Low (<1 ms)	Low	Low	Limited	Fully Deployable	High	Lower adaptability to novel attacks
[61]	CNN–LSTM Hybrid	Medium (5–50 ms)	High	Low	High	Deployable with optimization	Medium	Accuracy vs. computational cost
[96]	VehicBERT/ Attention Models	High (>50 ms)	Very High	Low	Very High	Limited	Medium	High memory and latency overhead
[103]	Temporal GNN	Medium (10–30 ms)	High	Low	High	Partially Deployable	Medium	Graph processing complexity
[109]	FedAvg-CNN	Medium	Medium–High	Very High	High	Deployable	Very High	Communication synchronization overhead
[115]	Multi-Agent RL	Adaptive/Variable	High	Low	High	Partial Deployment	Medium	Training convergence complexity
[121]	GAN-Based IDS	High	Very High	Low	Very High	Limited	Medium	Training instability and resource demand
[63]	RF + SVM + DT	Low–Medium	Medium	Low	Moderate	Fully Deployable	High	Increased model orchestration overhead

**Table 31:** Blockchain-based IDS logging mechanisms.

Ref.	Method	Application	Result and Benefit	Limitation
[168]	Blockchain + Feature Engineering	6G Vehicular IDS	High detection accuracy and tamper-proof logging	Storage overhead
[169]	Blockchain + Deep Learning	Trusted Execution IDS	Improved detection reliability and secure logging	High cost
[170]	Quantum DL + Ethereum Blockchain	Cloud/IDS Security	Enhanced fraud detection and strong integrity	High latency
[171]	Blockchain Risk Framework	Security management systems	Improved auditability and transparent logging	Scalability issues

**Table 32:** Blockchain-enhanced vehicular IDS architectures.

Ref.	Method	Blockchain Type	IDS Function	Result	Limitation
[164]	FL + Blockchain	Permissioned	Anomaly detection	Improved privacy and accuracy	Communication overhead
[165]	Blockchain-based IDS	Consortium	Collaborative detection	Enhanced detection rate	Consensus delay
[166]	Blockchain + Edge Intelligence	Distributed	Secure data sharing	Privacy preservation	Latency
[167]	Blockchain IoV Framework	Hybrid	Threat intelligence sharing	Improved scalability	Energy and latency trade-off

**Table 33:** Data scarcity challenges in vehicular IDS.

Ref.	Method	Dataset Type	Result	Challenge	Limitation
[172]	Industrial dataset analysis	Real + simulated	Limited generalization	Lack of real attack data	Dataset scarcity
[173]	Lightweight ML IDS	Controlled dataset	High accuracy	Dataset dependency	Poor real-world robustness
[174]	Survey-based evaluation	Mixed datasets	Identified the sim-reality gap	Model generalization failure	Limited real validation
[175]	Sequential AI IDS	Synthetic + real	Improved detection	Class imbalance	Missing attack types

**Table 34:** Real-time IDS constraints.

Ref.	Method	Technique	Result	Benefit	Limitation
[176]	Lightweight IDS	Feature engineering	Reduced latency	OBU-compatible	Slight accuracy loss
[177]	Hyper-dimensional computing	Memory-efficient model	Fast detection	Low latency	Scalability issues
[178]	Hybrid stacking model	Ensemble learning	High accuracy	Robust detection	High computation
[179]	Hybrid AI IDS	Optimization trade-off	Balanced performance	Efficient models	Complexity

**Table 35:** Scalability challenges in vehicular IDS.

Ref.	Method	Environment	Result	Benefit	Limitation
[180]	Real-time anomaly detection	Urban traffic	Improved scalability	Load balancing	RSU saturation
[181]	Collaborative IDS	Distributed VANET	High detection rate	Scalable architecture	Communication overhead
[178]	Hybrid IDS model	High mobility	Robust detection	Adaptive model	Performance variation

**Table 36:** Privacy trade-offs in vehicular IDS.

Ref.	Method	Privacy Technique	Result	Benefit	Limitation
[182]	Privacy-aware AI IDS	Data protection framework	Secure detection	Privacy preservation	Complexity
[183]	AI-driven IDS	Decentralized approach	Improved security	Reduced data exposure	Scalability
[184]	AI communication system	Secure V2V design	Enhanced safety	Data protection	Implementation cost

**Table 37:** Open challenges in vehicular IDS.

Ref.	Method	Focus Area	Result	Benefit	Limitation
[186]	Interoperability framework	System integration	Improved compatibility	Standardization support	Early-stage
[187]	Blockchain IDS framework	IoV security	Enhanced coordination	Secure architecture	Lack of standards

**Table 38:** Key challenges in adversarial robustness in vehicular IDS.

Ref.	Method	Attack Type	Result	Benefit	Limitation
[185]	Robust ML IDS	Adversarial attacks	Improved resistance	Strong defence	High cost
[188]	ML IDS evaluation	Evasion attacks	Vulnerability exposed	Insightful analysis	Weak robustness
[189]	RL-based IDS	Adaptive attacks	Improved defence	Adaptive learning	Latency

**Table 39:** Comparative analysis of adversarial attacks and defense strategies in vehicular IDS.

Ref.	Methodology	Attack Type	Defence Strategy	Dataset/Scenario	Performance Outcome	Limitations
[33]	Adversarial vulnerability analysis of IDS	Evasion attacks	Vulnerability evaluation	In-vehicle network IDS	Significant performance degradation under attack	No concrete defence mechanism
[76]	Adversarial attacks on supervised ML IDS	Gradient-based adversarial attacks	Basic adversarial testing	ML-based IDS models	Reduced detection accuracy	Lacks robust mitigation strategy
[185]	Robust ML-based IDS	Adversarial evasion attacks	Adversarial training	Vehicular network datasets	Improved robustness and detection stability	High computational overhead
[188]	Evaluation of adversarial robustness	Evasion and poisoning attacks	Robustness testing frameworks	Network intrusion datasets	IDS highly vulnerable without defence	No real-time vehicular validation
[189]	Reinforcement learning-based IDS	Adaptive adversarial attacks	RL-based adaptive defence	Simulated IDS environment	Improved adaptive attack resistance	High latency
[189]	Automated adversarial traffic generation	Traffic-based adversarial attacks	Synthetic attack generation	Network traffic simulation	Reveals IDS performance degradation patterns	Limited vehicular-specific validation

## Data Availability

No new data were created or analyzed in this study. Data sharing is not applicable to this article.
